# A Systematic Review of Folate and the Human Enteric Microbiome: Biological Mechanisms and Clinical Implications

**DOI:** 10.3390/ijms27115048

**Published:** 2026-06-03

**Authors:** Raunak Khanduja, Richard E. Frye

**Affiliations:** 1 The School of Clinical Medicine, University of Cambridge, Cambridge CB2 0SP, UK; rk661@cam.ac.uk; 2 Autism Discovery and Treatment Foundation, Phoenix, AZ 85020, USA

**Keywords:** folate, vitamin B9, microbiome, microbiota

## Abstract

Folate (vitamin B9) is central to one-carbon metabolism, supporting nucleotide biosynthesis, methylation homeostasis, and epigenetic regulation. The gut microbiome both produces and consumes folate, creating a bidirectional axis influencing host health and disease. We systematically reviewed 159 original studies from MEDLINE, Google Scholar, Embase, and Scopus (inception through January 2026) examining enteric microbiota–folate interactions, with intervention evidence graded using the Oxford Centre for Evidence-Based Medicine 2011 framework. Only a minority of gut bacteria possess complete folate biosynthetic pathways; most depend on cross-feeding from prototrophic taxa including *Bifidobacterium*, *Lactobacillus*, and *Streptococcus*. Altered microbial folate metabolism was associated with metabolic, gastrointestinal, oncologic, neuropsychiatric, cardiovascular, immunologic, and reproductive disorders through convergent mechanisms of disrupted methylation, genomic instability, and immune dysregulation. Probiotic interventions achieved the strongest evidence, supported by multiple human controlled and observational trials and animal models. The evidence for prebiotic, dietary, and folate supplementation interventions was moderate due to the predominant animal models and in vitro data. Overall, the predominant associational and observational evidence base is insufficient to establish causal relationships, underscoring the need for adequately powered human randomized controlled trials with folate-specific endpoints, multi-omics integration, and precision approaches matching folate form and dose to individual microbiome and host genetic profiles.

## 1. Introduction

Folate (vitamin B9) is a water-soluble vitamin essential for one-carbon metabolism, which underpins deoxyribonucleic acid (DNA) and ribonucleic acid (RNA) synthesis [1], methylation reactions [2], neurotransmitter production [2], and cellular growth and repair [3]. Folate deficiency is implicated in a range of clinical conditions, including megaloblastic anemia [4], cognitive impairment [5], depression [6], and pregnancy complications [7] such as neural tube defects (NTD) [4]. Since humans cannot synthesize folate de novo [8], adequate dietary intake from leafy green vegetables, legumes, fruits, and organ meats [9] or supplementation with folic acid is necessary [10]. The bioavailable folate intake during periconceptional periods is critical to reduce neural tube defect risk [11].

The human gut microbiome comprises a complex ecosystem that influences nutrient absorption [12], immune regulation [13], and metabolic function [14]. Of particular importance is the capacity of selected gut microbial species to synthesize folate de novo, thereby contributing substantially, although variably, to the host folate pool [15]. Conversely, dietary folate status and folic acid supplementation alter the composition, diversity, and function of the gut microbiome, affecting not only host nutrition but also local and systemic metabolic signaling [16].

This bidirectional interaction between folate metabolism and the gut microbiome is emerging as a critical axis in health and disease [17]. In multiple diseases, both folate metabolism and gut microbial profiles are perturbed, implicating host–microbiome crosstalk in their pathophysiology. Research suggests that manipulating the microbiome may improve folate availability and metabolic balance, opening new avenues for preventative and therapeutic interventions [15,18,19].

This systematic review aims to synthesize recent findings on microbial biosynthesis, the influence of folate status on microbiome composition and function, with an emphasis on recent clinical and translational insights. To our knowledge, this is the first review to take this comprehensive approach. The review will start off with a summary of folate biology and then discuss folate synthesis by the microbiome. Identified studies are divided into in vitro, in silico, non-diseased individuals, disease-specific studies, and intervention studies.

### 1.1. Folate Sources and Forms

**Figure 1:**
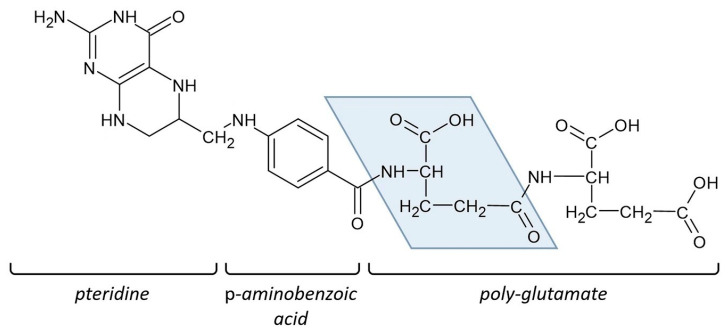
Chemical structure of folate. The molecule has three parts, the pteridine ring, the p-aminobenzoic acid and the glutamate tail. The glutamate tail can have several glutamate molecules attached to make a polyglutamate tail. Modified from [20].

Vitamin B9 (folate) refers to a family of chemically related molecules that share a common core structure, including natural folates and synthetic folic acid. These molecules feature a pteridine ring, which may exist in either reduced or oxidized states, connected to a p-aminobenzoic acid bridge and a mono- or polyglutamate tail of varying length (see Figure 1).

Folate occurs in multiple forms and is obtained primarily through the diet, largely as reduced polyglutamates found in leafy green vegetables, legumes, and fruits [9], but also as synthetic folic acid, an oxidized monoglutamate form used in supplements and food fortification [15]. Although both dietary folate and folic acid serve as precursors for active metabolites, their absorption and metabolic fates differ, with implications for bioavailability and physiological activity [20].

Folic acid is the synthetic form of folate which does not occur in nature. This is a critical concept as folic acid cannot be used by the body until it is reduced to tetrahydrofolate (THF) through dihydrofolate reductase (DHFR). DHFR has a very variable and limited capacity of about 331 µg per day in humans [21]. One study found that 200 µg added to a normal fortified diet was the upper limit at which unmetabolized folic acid appeared in the blood [22]. A double-blind, placebo-controlled (DBPC) study of older Chinese adults demonstrated that more than 800 µg of folic acid did not increase 5-methyl-THF (5-MTHF), the predominant biologically active form of folate, or reduce homocysteine [23]. Furthermore, high intake of folic acid can result in “pseudo Methylenetetrahydrofolate reductase (MTHFR)” syndrome characterized by elevated homocysteine, presumably due to UMFA inhibiting MTHFR [24]. Thus, it is very important to make the distinction between folic acid and folates that are more innate to the folate cycle such as 5-MTHF, THF and folinic acid (i.e., 5-formyltetrahydrofolate).

### 1.2. Microbiome-Driven Folate Synthesis

**Figure 2:**
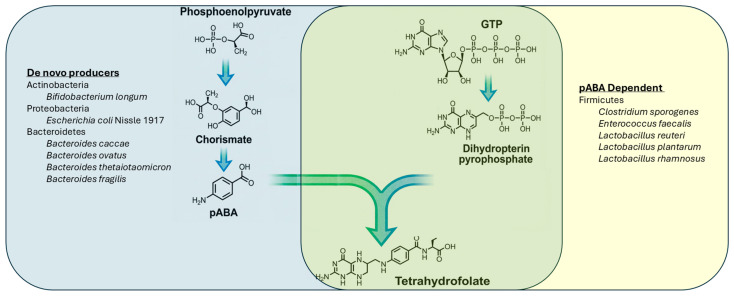
An overview of microbial folate synthesis. Prototrophs produce folate either de novo or when supplied with pABA. Examples of Phylum and strains contained in the human microbiome are provided. GTP: guanosine triphosphate; pABA: para-aminobenzoic acid. The figure was created in Microsoft PowerPoint and edited in Microsoft Paint.

The gut contains roughly 10^13^ to 10^14^ bacteria, comparable to the average number of human body cells [25]. The gut microbiota of mammals plays a key role in maintaining folate homeostasis [15]. Many gut bacteria, including *Bifidobacterium* and *Lactobacillus*, synthesize folate, either de novo or, when provided para-aminobenzoic acid (pABA) (see Figure 2). However, other bacteria may lack the ability to biosynthesize it, leading to a dependence on exogenous folate and therefore effectively become folate consumers [26]. Microbe-derived vitamins, including folate, can also be released into the intestinal environment during bacterial cell lysis, making folate from dying bacteria an additional source available to other microbes and the host [27]. This balance between folate-producing prototrophs (bacteria capable of de novo folate synthesis) and folate-dependent auxotrophs (bacteria that require exogenous folate or intermediates) contributes to folate homeostasis in the host.

The enteric microbiome includes complete or incomplete folate synthesizers (producers). The de novo folate-producing pathway in the human gut microbiome is a multi-step biosynthetic route involving two parallel pathways which converge on the production of THF (Figure 2).

The folate ring assembly is produced by converting chorismate, a substance which is produced from carbohydrates via the shikimate pathway, to pABA by chorismate lyase. Next, pABA synthase forms the folate ring from aminodeoxychorismate lyase and pABA.

Parallel to this, guanosine triphosphate (GTP) cyclohydrolase I, together with downstream enzymes, catalyzes conversion of GTP into dihydroneopterin, which is then metabolized to 6-hydroxymethyl-7,8-dihydropterin pyrophosphate (DHPPP). DHPPP is then joined with pABA by dihydropteroate synthase to form 7,8-dihydropteroate. Dihydrofolate synthase adds a glutamate residue to produce dihydrofolate (DHF). Finally, DHFR reduces DHF to THF, the active coenzyme used in one-carbon metabolism.

### 1.3. The Role of the Gastrointestinal Tract in Folate Supply

The gastrointestinal (GI) tract acquires folate through two fundamentally distinct processes. Exogenous dietary absorption occurs predominantly in the proximal small intestine while endogenous microbial biosynthesis is concentrated in the large intestine. These complementary folate sources contribute to whole-body folate status.

**Figure 3:**
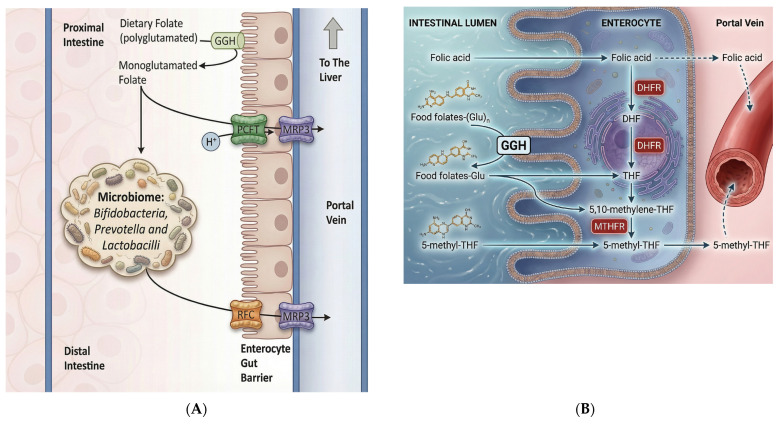
Absorption of folate. (**A**) Transport of folate from the gut lumen into the portal vein. (**B**) Metabolism of folate in the enterocytes. Abbreviations: GGH: γ-glutamyl hydrolase; PCFT: proton-coupled folate transporter; RFC: reduced folate carrier; MRP3: multidrug resistance-associated Protein 3; DHF: dihydrofolate; THF: tetrahydrofolate; DHFR: dihydrofolate reductase; MTHFR: methylenetetrahydrofolate reductase; (Glu)n: polyglutamated folates; Glu: monoglutamate folate. Adapted from [33,34]. Figures were created in Microsoft PowerPoint, graphically enhanced with Adobe Firefly and edited in Microsoft Paint.

Dietary folates exist as polyglutamated derivatives that require enzymatic hydrolysis in the gut. In the proximal small intestine, these polyglutamates are hydrolyzed to the absorbable monoglutamate form by γ-glutamyl hydrolase (GGH)—a step required for uptake across the enterocyte membrane (Figure 3A) [20]. Folic acid, due to its monoglutamate structure, bypasses this hydrolysis, enabling nearly complete absorption when ingested on an empty stomach [28]. However, folic acid first needs to be reduced at the nitrogen-8 position to form dihydrofolate (DHF), with further reduction at the nitrogen-5 position to yield THF (Figure 2). Natural food folate, by contrast, is biologically active without enzymatic reduction but only approximately 50% bioavailable due to incomplete deconjugation and matrix effects from the food source [29].

The uptake of folate and folic acid is mediated by specialized membrane transporters. The proton-coupled folate transporter (PCFT/SLC46A1) is the primary mechanism for folate absorption in the proximal small intestine (duodenum and jejunum), while the reduced folate carrier (RFC/SLC19A1) functions in the lower small intestine (ileum; Figure 3A) and the large bowel [30]. THF and natural folates receive methyl groups in the enterocyte by MTHFR, forming 5-MTHF, the predominant biologically active and circulating form in human plasma (Figure 3B) [20].

After formation, 5-MTHF is transported from the enterocyte into the portal circulation by multidrug resistance-associated proteins (Figure 3B). Folate is then distributed to peripheral tissues, where it can be taken up by various transporters (e.g, RFC, PCFT) or via folate receptor-mediated endocytosis [31]. In the liver, folate is stored in polyglutamate forms through the activity of folylpoly-γ-glutamate synthetase (FPGS). It is subject to enterohepatic recirculation, as a portion is secreted in bile and subsequently reabsorbed [32].

Although the small intestine accounts for the majority of daily folate uptake under physiologically replete conditions, the colonic microbiome constitutes a substantial endogenous folate depot. Genomic analyses of 512 representative GI bacterial genomes demonstrate that approximately 13% harbor complete de novo folate biosynthesis pathways, with an additional 39% able to synthesize folate when pABA is available from dietary sources or microbial cross-feeding [35].

Fecal folate concentrations exceed dietary intake, implying net luminal production. Direct evidence for colonic folate absorption was established through infusion of [13C]glutamyl-5-formyl-THF into the cecum, demonstrating labeled 5-MTHF appearance in plasma at 0.6 ± 0.2 nmol/h [36]. Subsequent delivery of enteric-coated [13C]-5-formyl-THF capsules past the ileocecal junction confirmed 46% apparent bioavailability of a physiologic dose delivered to the colon, attributable to the 20-fold longer colonic residence time [37].

Carrier-mediated folate transport at both apical and basolateral membranes of human colonocytes operates through a pH-dependent, DIDS-sensitive, electroneutral RFC-mediated process at luminal pH 4–5, distinct from the PCFT-dominated mechanism of the small intestine [38].

Quantitative estimates suggest colonic bacterial folate synthesis contributes approximately 322–396 µg of absorbable folate per day—an amount approaching or exceeding the adult RDA of 400 µg DFE [39]. While small intestinal absorption is intermittent and meal-dependent, microbial synthesis proceeds continuously, providing a steady-state luminal supply that stabilizes systemic folate homeostasis between meals [15,35,36].

Studies in piglets, which closely model human intestinal folate physiology, estimate that at least 18% of total folate requirements can be met through bacterial biosynthesis alone [40,41]. This figure is likely an underestimate in individuals consuming high-fiber diets that selectively enrich folate-producing genera such as *Bifidobacterium* and *Lactobacillus* [42]. An important mechanistic caveat is that the predominant luminal folate forms in the colon are polyglutamylated species released by bacterial lysis, which require deconjugation by colonocyte-expressed GGH prior to mucosal transport [35].

The magnitude of microbial folate contribution is highly variable between individuals and sensitive to microbiome composition, dietary fiber and prebiotic intake, antibiotic exposure, and probiotic supplementation. These factors collectively modulate the abundance and activity of folate-producing taxa and, consequently, the size of the colonic folate depot. Taken together, the current evidence supports a model in which dietary absorption in the small intestine provides the primary, kinetically rapid, and meal-contingent route of folate acquisition, while microbially derived colonic folate functions as a substantial, continuously replenished, and variable reserve whose contribution to systemic and colonocyte folate pools is underappreciated in standard dietary reference intake calculations. Colonic folate production may be clinically relevant in states of small intestinal disease, antibiotic-associated dysbiosis, or marginal dietary folate intake.

### 1.4. Consequences of Abnormal Folate Metabolism

To complement our discussion regarding diseases associated with folate metabolism, the pathological processes that drive them are reviewed here.

#### 1.4.1. Impaired One-Carbon (1C) Metabolism

Folate functions as the obligate carrier of one-carbon units that fuel three interdependent cytoplasmic biosynthetic pathways, and disruption of this central network propagates downstream across nucleotide synthesis, methylation, and amino acid homeostasis [43,44]. Depletion of 10-formyl-THF curtails donation of the C2 and C8 carbons essential for de novo purine ring assembly, reducing production of ATP, GTP, AMP, and GMP. This compromises energy metabolism, DNA and RNA synthesis, and proliferation in rapidly dividing tissues such as hematopoietic progenitors, GI epithelium, and embryonic cells [45,46]. Concurrently, methylene-THF deficiency impairs the reductive methylation of dUMP to dTMP via thymidylate synthase (TYMS), expanding the dUTP pool and driving uracil misincorporation into both nuclear and mitochondrial DNA. The resulting futile base-excision repair cycles generate single- and double-strand breaks, micronuclei, and overt chromosomal instability, with mitochondrial DNA bearing disproportionate vulnerability [1,46,47]. Finally, inadequate 5-MTHF availability blocks methionine synthase (MTR)-mediated remethylation of homocysteine to methionine, simultaneously producing hyperhomocysteinemia, hypomethioninemia, and depletion of SAM, the universal methyl donor [1,48,49,50].

#### 1.4.2. Disrupted Methylation Homeostasis

The reduction in methionine availability limits SAM synthesis and global methylation capacity, while SAH accumulation inhibits virtually all methyltransferases [51,52]. DNA hypomethylation is known to activate proto-oncogenes and derepress retrotransposons in cancer [53], and SAM-dependent histone methylation at H3K4, H3K9, and H3K27 is similarly dysregulated [54], reprogramming chromatin architecture [55]. Paradoxically, supraphysiologic folic acid inhibits DHFR, causing DHF accumulation that secondarily inhibits MTHFR, causing a functional “pseudo-MTHFR deficiency”, resulting in global hypomethylation, while hypermethylating DNA repairs gene promoters, elevating de novo point mutations through a distinct mechanism [8].

#### 1.4.3. Genomic Instability

Folate insufficiency promotes genomic instability by depleting thymidylate and increasing the dUTP/dTTP ratio, driving uracil misincorporation into DNA with consequent base-excision-mediated strand breaks and chromosomal fragility [56]. Genomic uracil levels are approximately 8–9-fold higher in deficient versus replete individuals, with an estimated four million uracil misincorporations per cell under severe depletion [46]. Micronuclei and nuclear anomalies are directly observed in human lymphocytes under low-folate conditions, with certain instability endpoints showing greater increases in MTHFR 677TT cells [57]. During S-phase, sumoylation-dependent nuclear import of the TYMS-SHMT-DHFR complex is required for in situ dTMP biosynthesis adjacent to replication forks [58].

#### 1.4.4. Hematologic Consequences

Impaired purine and thymidylate synthesis in folate or B12 deficiency cripples DNA replication in hematopoietic precursors, causing megaloblastic anemia with macrocytic erythrocytes, hypersegmented neutrophils, and multilineage cytopenias [59]. Both deficiency and supraphysiologic folate compromise B-lymphocyte proliferation and marrow reconstitution, underscoring the nonlinear dose response in hematopoiesis [60].

#### 1.4.5. Neurological Consequences

5-MTHF is the dominant folate species in cerebrospinal fluid, required for myelin synthesis [61], monoamine neurotransmitter production [62], and neuronal proliferation [63]. Impaired methylation of myelin basic protein contributes to white matter abnormalities. Folate supports BH_4_ regeneration, an essential cofactor for dopamine, serotonin, and noradrenaline hydroxylases [64]; deficiency reduces neurotransmitter output, linking one-carbon metabolism (OCM) to neuropsychiatric phenotypes [65]. During the embryogenic window (days 21–28), deficient nucleotide synthesis underlies failed neural tube closure [66]. Across the lifespan, 5-MTHF insufficiency manifests as cognitive dysfunction, depression, schizophreniform psychosis, ataxia, and neurodevelopmental regression [67,68], compounded by homocysteine neurotoxicity through NMDA receptor overstimulation [69].

#### 1.4.6. Cardiovascular and Thrombotic Consequences

Hyperhomocysteinemia impairs eNOS signaling and NO bioavailability via ADMA accumulation, augments vascular oxidative stress, and drives lipid peroxidation, LDL oxidation, and foam cell formation [70]. Homocysteine induces vascular smooth muscle proliferation through p38 MAPK/p47phox signaling [71], while epigenetic silencing of extracellular superoxide dismutase (SOD) by promoter hypermethylation further amplifies oxidative vascular injury, establishing a self-reinforcing atherogenic cycle [72].

#### 1.4.7. Carcinogenesis

Cancer risk from abnormal folate metabolism arises from the convergence of genomic instability and disrupted methylation. Deficiency destabilizes the genome through uracil misincorporation while creating a permissive epigenetic landscape via global hypomethylation and promoter hypermethylation. This relationship is nonlinear: deficiency promotes initiation, whereas excess accelerates pre-existing neoplasms [73].

#### 1.4.8. Mitochondrial Dysfunction

Approximately 40% of cellular folate resides within mitochondria, supporting formate export to cytoplasmic folate pathways and in-organelle dTMP synthesis [74]. Mitochondrial DNA is more susceptible to uracil misincorporation than nuclear DNA due to the absence of histones, ROS proximity, and limited repair capacity [75]. Uracil-driven mtDNA strand breaks trigger leakage of mtDNA fragments into the cytoplasm, activating cGAS-STING innate immune signaling and apoptotic cascades [76].

## 2. Materials and Methods

A systematic review of the scientific literature was conducted to investigate the interplay between folate (vitamin B9) and the human gut microbiome, with a particular focus on mechanisms, clinical implications, and potential therapeutic interventions. The protocol was retrospectively registered at the Center for Open Science (https://doi.org/10.17605/OSF.IO/BC8ZH) [77].

### 2.1. Literature Search Strategy

A computer-aided search of MEDLINE, Google Scholar, Embase, and Scopus, from inception through 25 January 2026 was conducted to identify pertinent publications. The search utilized combinations of keywords and Medical Subject Headings (MeSH) related to “folinic”, “leucovorin”, “folate”, “folic”, “methylfolate”, “5MTHF”, “levofolinic”, “folinate”, “formyltetrahydrofolate”, “vitamin B9,” “5-methyltetrahydrofolate,” combined with “microbiota”, “microbiome”, “gut flora” and “gut bacteria.” The references cited in identified publications were also searched to locate additional studies.

### 2.2. Inclusion/Exclusion Criteria

Eligible publications included English-language original research articles. Articles that did not present new or unique data such as review articles or letters to the editor were excluded. Articles that did not specifically examine both changes in the gut microbiome and folate were also excluded. Studies that only used folate as a marker or examined used bacteria as part of an assay were excluded. Studies that tested toxins, antibiotics, infections, vaccination or cancer treatments such as chemotherapy, methotrexate (MTX) or radiation were excluded, as such interventions affect both folate and the microbiome separately. Also, studies which used mixed supplementation that included folate were excluded.

### 2.3. Study Selection and Assessment

**Figure 4:**
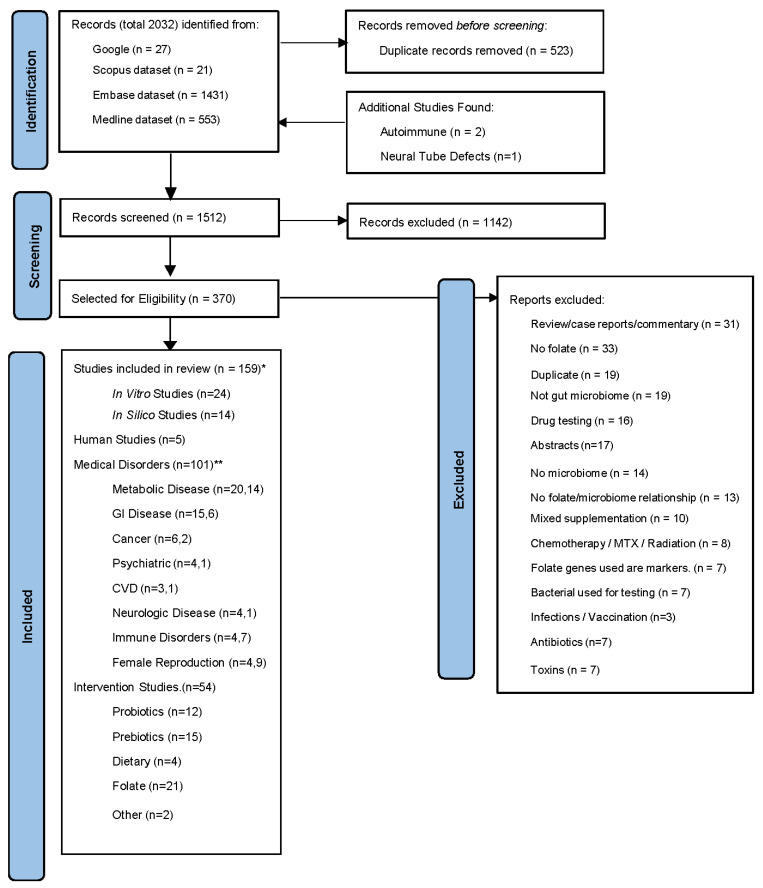
PRISMA diagram. * Some studies are overlapping, so the number of studies in each subsection adds up to more than the total number of studies. ** Categories: numbers in parentheses represent the number of descriptive studies and number of intervention studies. Abbreviations: CVD: cardiovascular disease; GI: gastrointestinal; MTX: methotrexate.

This systematic review followed PRISMA guidelines [78] and the PRISMA Checklist is found in Supplementary Table S1. The PRISMA Flowchart is displayed as Figure 4. To mitigate potential intellectual or selection bias, study screening, eligibility assessment, and data extraction were performed independently and in duplicate by two reviewers using pre-specified eligibility criteria registered prospectively on OSF; disagreements were resolved by consensus. Both authors independently examined each identified study in depth and assessed factors such as selection, performance detection, attrition, and reporting biases as per standardized guidelines [79].

### 2.4. Synthesis of Results

Given the heterogeneity in design, population, and outcomes of included studies, a quantitative meta-analysis was not considered appropriate; therefore, a narrative synthesis approach was adopted. Identified studies were grouped thematically by study type into in vitro, in silico, non-diseased individuals, disease-specific studies, and intervention studies. Disease-specific studies were further categorized broadly into metabolic disease, gastrointestinal disorders, cancer, psychiatric disease, cardiovascular disease, neurologic disease, immune disorders and female reproduction. Within each group, findings were summarized descriptively, with attention to the direction and consistency of reported effects. Where relevant, study quality and methodological limitations were considered in the interpretation of findings.

### 2.5. Evidence Quality Assessment

A GRADE-style certainty assessment was applied across all studies reviewed in Section 3 to provide a consistent statement of confidence in the evidence across mechanistic, observational, animal, and interventional domains. Human randomized or quasi-randomized trials were considered to start at high certainty since these designs are least susceptible to confounding when well conducted and are most direct for treatment–effect questions. Human observational studies start at low certainty since these designs are more vulnerable to confounding, selection bias, and reverse causality. All non-human or mechanistic studies, including animal, in vitro, ex vivo fecal slurry, and in silico studies, are very low certainty because of substantial indirectness to human clinical questions. Certainty was then downgraded where appropriate for risk of bias, inconsistency, indirectness, imprecision, and suspected publication bias, and upgraded only in uncommon cases where a large effect, dose–response relationship, or particularly coherent triangulation across methods strengthened confidence in the finding (see Supplementary Table S2). Because many studies in this field rely on small sample sizes, cross-sectional designs, inferred microbial pathway analysis, or mechanistic models, most non-human studies remain very low certainty and most human observational studies remained low certainty after assessment. Studies defined as high certainty are those that we can be very confident that the true effect is close to the estimated effect. Moderate certainty means that the true effect is probably close to the estimated effect, but there is a possibility that it is substantially different. Low certainty means that the true effect may be substantially different from the estimated effect; and very low certainty means that the true effect is likely to be substantially different from the estimated effect.

### 2.6. Risk-of-Bias Assessment

The risk of bias of every included study was independently rated using a design-appropriate tool (see Supplementary Table S2). Randomized controlled trials of probiotic, prebiotic, dietary, or folate interventions in humans were assessed with the Cochrane Risk of Bias 2 (RoB 2) tool across its five domains (randomization process; deviations from intended interventions; missing outcome data; measurement of the outcome; selection of the reported result), with an overall judgment of low risk, some concerns, or high risk. Non-randomized human intervention studies (e.g., open-label probiotic, dietary, or supplementation studies) were assessed with the ROBINS-I tool across its seven domains (confounding; selection of participants; classification of interventions; deviations from intended interventions; missing data; measurement of outcomes; selection of the reported result), yielding overall judgments of low, moderate, serious, or critical risk. Observational human studies (cohort, case-control, and cross-sectional designs) were assessed with the Newcastle-Ottawa Scale (NOS), generating a star rating across the selection, comparability, and outcome (or exposure) domains and an overall judgment of good (≥7 stars), fair (5–6 stars), or poor (<5 stars). Animal experimental studies were assessed with SYRCLE’s Risk of Bias tool, which mirrors RoB 2 but adds animal-specific items including sequence generation, baseline characteristics, allocation concealment, random housing, blinding of caregivers, random outcome assessment, blinded outcome assessment, incomplete outcome data, selective reporting, and other potential sources of bias. For in vitro, in silico, ex vivo, computational, and mechanistic studies, which fall outside the scope of standard clinical bias tools, a structured narrative methodological appraisal was applied that documented strain or genome characterization, analytical reproducibility, donor or community variability, and the ecological or biological generalizability of the design.

Domain-level judgments were grounded in characteristics that were retrievable from the original reports; where allocation, blinding, attrition, or selective reporting could not be ascertained, conservative defaults of “some concerns” (RoB 2) or “moderate” (ROBINS-I, SYRCLE) were applied and explicitly flagged. The complete study-level risk-of-bias assessment is provided in Supplementary Table S2. Section-level summaries in Section 3 below incorporate the dominant risk-of-bias judgment for each evidence category alongside the GRADE certainty rating.

### 2.7. Levels of Evidence for Intervention Studies

The quality of evidence for intervention studies was assessed using the Oxford Centre for Evidence-Based Medicine (OCEBM) 2011 Levels of Evidence framework (OCEBM Levels of Evidence Working Group, 2011 [80]). This framework classifies individual studies into five levels based on study design and methodological rigor for questions of treatment benefit: Level 1 (systematic reviews of randomized trials or n-of-1 trials), Level 2 (randomized trials or observational studies with dramatic effect), Level 3 (non-randomized controlled cohort/follow-up studies), Level 4 (case-series, case-control, or historically controlled studies), and Level 5 (mechanism-based reasoning). Each intervention category was then assigned an overall grade of recommendation: Grade A (consistent Level 1 studies), Grade B (consistent Level 2 or 3 studies, or extrapolations from Level 1 studies), Grade C (Level 4 studies or extrapolations from Level 2 or 3 studies), or Grade D (Level 5 evidence or troublingly inconsistent or inconclusive studies of any level). Levels may be downgraded based on study quality, imprecision, indirectness, or inconsistency between studies, and may be upgraded if there is a large or very large effect size.

## 3. Results

159 articles were selected for review (Figure 4). Of these, 83 were disease-specific, most of which examined metabolic disease and GI disorders. Few studies examined cancer, cardiovascular, psychiatric, neurologic and immune disorders and female reproduction. Five non-intervention studies examined non-diseased humans. Fifty-four studies examined interventions. The intervention studies examined humans and animal models in both health and disease states.

### 3.1. In Vitro and In Silico Studies

To study microbiomes in the laboratory without an animal model, two approaches are used. Using in vitro studies, the completeness of microbial folate biosynthetic pathways is determined by using two primary approaches. The empirical approach measures folate production in bacterial cultures via sensitive microbiological assays or chromatography-based methods, or by examining growth of organisms in folate-free media [15,81]. In contrast, in silico studies use genomics-based approaches which rely on gene annotation and pathway reconstruction. The presence of critical genes for the four canonical folate modules (chorismate/shikimate pathway, pABA biosynthesis, pterin biosynthesis, folate assembly/polyglutamylation) are determined from sequenced genomes to identify strains possessing all, or a subset of, modules required for de novo folate synthesis [35]. If all required genes are present and predicted to be functional, the pathway is considered “complete” while organisms with missing or pseudogenes indicate partial or incomplete capability [82].

#### 3.1.1. In Vitro Studies

Across in vitro studies, folate appears as a central metabolite that structures gut-like microbial communities by distinguishing folate-producing “prototrophs” from folate-dependent “auxotrophs” and enabling nutrient cross-feeding. Folate-producing lactic acid bacteria such as *Lactiplantibacillus plantarum* LZ227 [83], *Latilactobacillus sakei* LZ217 [84], and *Lactobacillus reuteri* (human clade II and VI strains) [85], as well as classic *Bifidobacteria* including *Bifidobacterium catenulatum* ATCC 27539 [86], *Bifidobacterium animalis* subsp. *animalis* ATCC 25527 [86], *Bifidobacterium adolescentis* DSM 20083T [87] and ORG10 [88], *Bifidobacterium pseudocatenulatum* DSM 20438T [87], *Bifidobacterium pseudolongum* subsp. *Globosum* [88], and *Bifidobacterium dentium* CHZ9 [88], synthesize mainly reduced folate forms such as 5-MTHF and THF, often at high intracellular concentrations. In fecal or slurry fermentations under folate-free or low-folate conditions, strains like *L. plantarum* LZ227 [83] and *L. sakei* LZ217 [84] increase overall microbial diversity, enrich Bacillota and butyrate-producing genera (*Faecalibacterium*, *Ruminococcus* 2, *Butyricicoccus*, *Lactobacillus*), and suppress Bacteroidota and potential pathobionts such as *Alistipes*, *Parabacteroides*, and *Bacteroides*, while shifting short-chain fatty acid (SCFA) profiles toward increased butyrate and reduced acetate. Another study found that the probiotic strain *Streptococcus thermophilus* IDCC 2201 was a major folate producer which was necessary for folate-dependent growth of *Bacteroides thetaiotaomicron*, *Veillonella parvula*, and *Ruminococcus faecis* [89].

Folate-producing gut commensals span multiple families, including Lactobacillaceae (*L. plantarum*, *L. reuteri*, *L. sakei, Pediococcus* spp.), Bifidobacteriaceae (*B. adolescentis*, *B. longum*, *B. pseudocatenulatum*), Streptococcaceae (*S. thermophilus*), and Bacillaceae (*Bacillus subtilis*) [15,35,84,85,86,87,88,89,90,91,92,93,94,95,96,97]. *S. thermophilus* IDCC 2201 was found to enhance folate biosynthesis when co-cultured with individual gut commensals [98]. Production varies substantially: some strains secrete 5-MTHF extracellularly for cross-feeding with non-producers such as *Roseburia intestinalis* and *Faecalibacterium prausnitzii*, while others retain folate intracellularly [87,99,100]. This metabolic interdependence drives community-level folate cycling and ecosystem stability [101]. Conversely, certain commensals—*Marvinbryantia formatexigens*, *Blautia hydrogenotrophica*, and *Eubacterium* hallii—are folate auxotrophs that consume microbial folate, competing with the host for this resource [99,102,103]. The net folate output available to the host depends on the balance between producers and consumers within a given microbial community [104,105].

Detailed liquid chromatography–mass spectrometry (LC–MS) analyses of *B. adolescentis* DSM 20083T and *B. pseudocatenulatum* DSM 20438T demonstrate that nearly all 5-MTHF synthesized in folate-free medium is retained intracellularly as polyglutamate (5-CH_3_-H_4_PteGlu_4_), with extracellular folate appearing in proportion to cell lysis rather than active secretion. This suggests that microbial folate primarily supports bacterial metabolism and interbacterial nutrient transfer, with host availability dependent on turnover and deconjugation [87]. Comparative work across hosts indicates that *Bifidobacteria* from primates (humans, chimpanzees, orangutans) are robust folate producers (e.g., *B. adolescentis* and *B. dentium*) with total folate often >7000 µg/100 g DM. In contrast, isolates from carnivores (dog, cheetah) and insects (honeybee) lack evident folate biosynthesis, pointing to a linkage between host phylogeny and microbial folate capacity [88].

A distinct line of experiments in the *Caenorhabditis elegans*–*Escherichia coli* model highlights that bacterial folate can also be detrimental for the host: inhibiting *E. coli* folate synthesis or reducing *E. coli* folate production with guarana extract extends *C. elegans*’ lifespan [102], whereas high *E. coli* folate accelerates aging via mechanisms independent of the worms’ folate status [103].

Together, these in vitro data depict folate as a pivotal microbial currency that does the following:

1. Allows specific taxa, such as *L. plantarum* LZ227, *L. sakei* LZ217, *S. thermophilus* IDCC 2201, *L. reuteri* strains, multiple *Bifidobacterium* species, *M. formatexigens*, *B. hydrogenotrophica*, *Blautia producta*, and several *Bacteroides* spp., to function as vitamin “hubs”.
2. Supports the growth of folate-auxotrophic butyrate producers like *R. intestinalis* and many *Lachnospiraceae* through cross-feeding.
3. Exerts context-dependent effects on the host—beneficial when it enhances butyrate-producing commensals and potentially harmful when excessive bacterial folate drives pro-aging or other adverse pathways.

#### 3.1.2. In Silico Studies

In the human enteric microbiome, a bacterium can be classified as a potential folate producer if its genome contains at least one set of genes involved in the folate biosynthetic pathway, including genes required for the synthesis of (1) chorismite, (2) pABA, (3) DHPPP, and (4) THF from pABA and DHPPP [15]. An evaluation of 512 human enteric bacterial genomes showed that 13% of bacteria contain genes spanning all four pathways, required for complete de novo THF synthesis. The remainder (>86%) require folate or folate intermediates (e.g., pABA or DHPPP) from other gut bacteria or the human diet [35]. For example, an additional 39% of bacterial genomes could produce THF with extra pABA provided [35]. An analysis of 256 human gut microbiome genomes showed that a majority of Bacteroidota (92%), Fusobacteriota (79%) and Pseudomonadota (71%) have the genes to synthesize folate de novo, whereas a minority of Actinomycetota (26%) and Bacillota (15%) are capable of de novo folate synthesis, as many of their genomes lack the pABA biosynthesis pathway [82].

In the human gut context, subsystems-based reconstructions over 2228 genomes representing 690 cultured species delineate complete, partial, and absent pathways for eight B-vitamins (B1, B2, B3, B5, B6, B7, B9, B12), allowing classification of species as folate prototrophs or auxotrophs and revealing conserved “vitamin phenotypes” at higher taxonomic levels [105]. These analyses emphasize that many bacteria carry only partial folate or other B-vitamin pathways and instead rely on uptake of intermediates (vitamers) such as thiazole, quinolinate, dethiobiotin, or pantoate, expanding the menu of potential syntrophic interactions. Complementary ruminant metagenomics across seven stomach species identify that only a minority can synthesize five or more vitamins, with many encoding only one [106]. Evolutionary genomics of *L. reuteri* shows human-adapted ecotypes differ in folate, vitamin B12, and arginine metabolism gene clusters, with an *AraC* family regulator (*PocR*) co-regulating folate with other metabolic and immunomodulatory traits, underscoring that folate synthesis is embedded in broader probiotic functional repertoires [85].

At the level of specific bacteria, multiple lactic acid and spore-forming taxa are highlighted as folate producers or as harboring defined folate-pathway genes. *L. plantarum* ZFM55, isolated from infant feces, carries 20 folate-synthesis genes and produces ~300 ng/mL folate in vitro. It increases *Bifidobacterium* abundance and suppresses *Escherichia* and *Shigella*, positioning it as a folate-producing probiotic candidate [91]. Human-derived *L. reuteri* strains (ATCC 55730 and ATCC PTA 6475), and more broadly *L. reuteri*, have genome-encoded folate pathways [92]. A more recent study identifies *L. reuteri* Lreu_1276 as encoding a structurally novel dihydroneopterin triphosphate pyrophosphohydrolase, a key step in THF biosynthesis, implying diverse, non-homologous enzyme support of folate pathways across intestinal bacteria [93].

Probiotics with predicted folate capacity include *Bacillus subtilis* DE111 [94] and *Bacillus clausii* B106 [95]. Two *Pediococcus acidilactici* strains (WNYM01 and WNYM02), isolated from human gut microbiota, have complete genome sequences with identified folate gene clusters [96]. *L. plantarum* NPL1378 mucosa-associated lactic acid bacteria from the human ileocecal region are genomically characterized as a folate-producing strain harboring stress-tolerance, adhesion, bile salt hydrolase, bacteriocin, and antioxidant genes [97]. One study found strong evidence for synthesis of folate in *R. inulinivorans* genomes but not in other *Roseburia* spp. [107].

Comparative genomics of poultry and swine *Lactobacillales*, including *Enterococcus lactis*, *Enterococcus mundtii*, *Ligilactobacillus agilis*, *L. reuteri*, and *Limosilactobacillus vaginalis*, shows heterogeneous but complementary distributions of folate, riboflavin, and menaquinone pathways [108].

Taken together, in silico analyses portray folate not as a uniformly distributed metabolite, but as a node in a distributed network of biosynthetic specialists (e.g., *L. plantarum* ZFM55, *L. reuteri* strains, *B. subtilis* DE111, *B. clausii* B106, *P. acidilactici* WNYM01/WNYM02, *E. lactis*, *E. mundtii*, *L. agilis*, *L. reuteri*, *L. vaginalis*, *L. paragasseri*, *L. plantarum* NPL1378) and auxotrophic consumers, with extensive sharing and salvage that help stabilize community structure under wide variation in dietary B-vitamin supply.

#### 3.1.3. GRADE Summary

The in vitro, ex vivo, and in silico studies consistently support the biological plausibility that folate production and auxotrophy shape microbial community structure, cross-feeding, and ecosystem stability. However, because these studies are mechanistic and indirect with respect to human clinical outcomes, they start at very low certainty and generally remain very low certainty after GRADE assessment (see Supplementary Tables S2.1 and S2.2). Accordingly, these studies provide important mechanistic support but not high-certainty clinical evidence. Across the in vitro and in silico literature, the structured narrative appraisal indicates predominantly moderate methodological risk, reflecting strong analytical reproducibility offset by limited donor or strain diversity and indirect host relevance (Supplementary Tables S2.1 and S2.2).

### 3.2. Folate–Gut Axis in Normal States of Health

In a cross-sectional analysis of 2111 Dutch children (9–12 years) and 1427 Dutch adults (46–88 years), children had a lower alpha diversity but a microbiome functionally enriched for folate biosynthesis, whereas adults showed relatively less predictable folate biosynthesis [109]. In an observational study of fecal cultures from 200 adults, higher folate production was linked to lower alpha diversity and higher relative abundance of *Bacteroides*, *Sutterella*, and *Parasutterella* [110].

In a case–control study, 25 endurance athletes were found to have a more diverse microbiota with higher *Prevotella* as compared to 46 sedentary controls. A positive association was found between folic acid intake and the family *Christensenellaceae* [111].

Following a 6-month sea voyage, 30 seafarers were found to have an increased Bacillota/Bacteroidota ratio, loss of *Bacteroides*, and decreased folate biosynthesis [112].

In 158 Kazakh adults spanning four frailty-severity categories, worse frailty was associated with lower gut diversity and Bacillota (including Clostridia and Erysipelotrichia) and higher Bacteroidia and Actinomycetota, along with higher folate microbiome biosynthesis [113].

The studies in non-diseased individuals show broadly consistent associations between microbial folate biosynthesis potential, diet, age, physiological state, and frailty, but most are cross-sectional and rely on inferred pathway-level functional analyses rather than direct flux measurements. These human studies therefore provide low-certainty evidence that microbial folate capacity is associated with normal physiological variation, while supporting mechanistic studies remain very low certainty because of indirectness (see Supplementary Table S2.3). Risk of bias on the Newcastle-Ottawa Scale was fair to good across these observational cohorts, with the principal limitations being incomplete dietary-intake adjustment and reliance on inferred microbial folate function (Supplementary Table S2.3).

### 3.3. Folate–Gut Axis in Disease States

In this section, studies supporting the association between folate abnormalities and the microbiome in human diseases are reviewed. Table 1 summarizes the disease categories and specific disorders, the abnormal folate-related mechanisms primarily driving the disease, and the downstream consequences of the disease state.

#### 3.3.1. Metabolic Disease

##### Obesity

Obesity may reflect chronic disturbances in energy and one-carbon metabolism, with excess adiposity accompanied by alterations in hepatic one-carbon pathways, homocysteine handling, and related epigenetic regulation. Folate status both responds to and modulates these changes: obesity may be associated with lower serum folate alongside higher red blood cell folate and altered folate handling, while low folate intake or deficiency may be linked to greater adiposity, enhanced adipocyte lipid accumulation, and higher obesity risk [114].

The microbiome associated with human obesity is characterized by reduced diversity, enrichment of proinflammatory taxa, and depletion of folate metabolism. This occurs alongside increased saccharolytic pathways, suggesting a possible shift away from microbial folate production and toward carbohydrate-driven, lipogenic metabolism [115]. In one study, *Parasutterella secunda*, *Butyrivibrio crossotus*, and *Clostridium saccharogumia* were significantly positively correlated with folic acid levels [116]. In pregnant overweight women, *Bacteroides* and *Bifidobacterium* abundances, which correlate positively with circulating folate, are depleted, linking loss of specific commensals to impaired host folate status [117]. Large cohort work also shows that folic acid intake and serum folate associate with particular phyla (e.g., Actinomycetota), tying dietary folate exposure to compositional shifts [118]. Finally, metagenomic and metatranscriptomic studies repeatedly identify folate biosynthesis/metabolism pathways as differentially enriched in obesity [119], positioning microbial folate pathways as a recurring functional signature of adiposity [115,116,117,118,119].

##### MASLD

Metabolic dysfunction-associated steatotic liver disease (MASLD, formerly NAFLD) arises from hepatic insulin resistance-driven excess free-fatty-acid influx, de novo lipogenesis, and lipotoxicity, which together may promote oxidative stress, mitochondrial dysfunction, and hepatocellular injury [120]. Folate, as a key one-carbon donor, is tightly linked to this process: lower dietary and serum folate may be associated with higher MASLD prevalence, and experimental and human data suggest that inadequate folate may impair hepatic lipid handling and promote steatosis and inflammation [121]. Disrupted folate metabolism in MASLD may disrupt methionine and one-carbon metabolism, alter DNA methylation and lipid-regulatory signaling (including PPARα pathways), and promote hepatic triglyceride accumulation and progression of steatosis [122].

Across MASLD studies, folate-related microbial metabolism consistently emerges as a key node in the gut–liver axis that shapes steatosis risk, severity, and response to interventions. Human metatranscriptomic work shows that Argentinian MASLD patients have enriched pathways for sulfur oxidation, SCFA, purine, and lipopolysaccharide (LPS) synthesis, whereas microbial folate synthesis is relatively enriched in simple steatosis compared with steatohepatitis, suggesting that loss of folate-producing capacity may accompany progression to more severe disease [123]. In those with IBD-associated NAFLD, increased *Lactococcus*, decreased *Coprococcus* 3, and *Ruminococcus* 2 was correlated with changes in folate and other vitamin B metabolic pathways [124]. Chicken steatosis was linked to low microbiome diversity, particularly alterations in duodenal *Lactobacillus* species, as well as reduce folate metabolism [125].

In adults with MASLD, case-control metagenomics reveals lower abundances of protective taxa (*Alistipes senegalensis*, *Coprococcus eutactus*, *Faecalibacterium*) and reduced expression of pathways for methionine, folate, and branched-chain amino acids (BCAA) metabolism, particularly in those consuming low-fiber, high-added-sugar, high-saturated-fat diets, linking Western dietary patterns to impaired microbial one-carbon metabolism [126]. Among people with HIV and MASLD, rectal microbiome and 16S-based profiling demonstrate that steatosis and fibrosis are associated with distinct dysbiosis and decreased folate biosynthesis pathways, with severity tied to reduced predicted folate production and other metabolic shifts [127,128,129].

These studies link increased folate production by duodenal *Lactobacillus* and altered one-carbon/methionine-cycle metabolites to hepatic steatosis, suggesting that microbiome-derived folate may interact with host methylation and phospholipid pathways to influence fatty liver development.

##### Diabetes Mellitus

Lower folate status and chronic folate deficiency may be associated with higher diabetes risk and, in experimental and human studies, could contribute to impaired glucose and lipid metabolism, hepatic steatosis, insulin resistance, and worse glycemic control, whereas higher folate intake may improve insulin sensitivity and reduce the incidence of diabetes [130].

Adolescents with type 1 diabetes show downregulation of folate, riboflavin, and biotin biosynthesis pathways with increased fermentation modules, indicating a shift away from vitamin production toward energy extraction [131].

In type II diabetes mellitus (T2D) mouse models, multiple interventions improve glycemia while modulating microbial folate pathways. A rapeseed meal polyphenol extract reshapes dysbiotic communities (increasing *Alistipes*, Bacteroidales, *Allobaculum*, *Parabacteroides*; reducing *Desulfovibrio* and *Oscillibacter*) and alters 150 metabolites, with folate biosynthesis among the significantly regulated pathways, alongside improved insulin sensitivity [132]. Similarly, a *Sanghuangporus vaninii* mixture shifts the microbiota toward Bacteroidota, Verrucomicrobia, *Akkermansia*, *Alloprevotella*, and *Blautia* and modulates Kyoto Encyclopedia of Genes and Genomes (KEGG) modules including folate biosynthesis, in parallel with better glucose/lipid indices and reduced inflammation [133].

##### Metabolic Disease Summary

The folate–microbiome axis may be dysregulated in metabolic disease through a coordinated loss of folate-producing commensals and metabolic reprogramming toward saccharolytic and lipogenic pathways. Reduced microbial folate biosynthesis capacity is consistent across obesity, MASLD, and diabetes, but contradictions persist regarding directionality, particularly the paradoxical pairing of low serum folate with elevated red blood cell folate in obesity, and locally enriched duodenal *Lactobacillus*-derived folate in MASLD. Human studies provide low-certainty evidence for an association between disturbed microbial folate metabolism and metabolic disease phenotypes, while animal and mechanistic literature remains very low certainty for direct clinical inference. Overall, the certainty that altered folate-related microbiome function plays a causal role in metabolic disease is low (see Supplementary Table S2.4). Priority research directions include longitudinal and interventional human studies that distinguish microbially produced from dietary folate, mechanistic dissection of how site-specific folate fluxes drive hepatic methylation and lipid handling, and trials testing whether restoring folate-producing taxa improves cardiometabolic outcomes. Risk of bias was fair on the Newcastle-Ottawa Scale for the human observational studies and moderate on SYRCLE for the supporting animal studies, with the dominant limitations being inferred-function analyses, incomplete confounder adjustment, and unclear allocation/blinding (Supplementary Table S2.4).

#### 3.3.2. Gastrointestinal Disorders

Gastrointestinal disorders may be particularly susceptible to abnormal folate metabolism given the high proliferative rate of intestinal epithelium. Defective nucleotide synthesis compromises mucosal renewal, while methylation imbalance may amplify immune activation and barrier dysfunction. Uracil misincorporation in rapidly dividing epithelial cells could further promote mucosal injury and neoplastic transformation.

##### Inflammatory Bowel Disease (IBD)

IBD is now understood as a chronic immune-mediated disorder with a strong metabolic component, in which Western-diet–driven excess of specific macronutrients, altered microbial metabolism, and disturbed cellular energy pathways may cooperate with genetic and immune dysregulation to sustain intestinal inflammation [134]. Folate, an essential one-carbon donor absorbed in the proximal small intestine, is frequently low in IBD and meta-analytic and mechanistic studies show that reduced serum folate in IBD is associated with folate deficiency, hyperhomocysteinemia, and worse inflammatory and thrombotic risk, suggesting a link between disturbed folate metabolism, intestinal inflammation, and disease course [135].

Prospective cohort work in healthy first-degree relatives of Crohn’s patients who later developed ulcerative colitis (UC) shows that preclinical microbiome configurations associated with future UC risk are enriched or depleted for specific metabolic pathways, including several involved in folate metabolism [136]. An IBD canine model demonstrated low serum folate along with a correlation between higher clinical activity scores and lower fecal *Lactobacillus* and total SCFA levels [137]. In Crohn’s disease, metagenomic analysis during exacerbations shows a broad depletion of microbial genes involved in biosynthesis of folate, as well as SCFA production [138]. In IBD-associated spondyloarthritis, sulfasalazine efficacy depends on a microbiome enriched in *F. prausnitzii* and butyrate production; sulfapyridine promotes butyrate synthesis in *F. prausnitzii* in vitro, but this is suppressed by excess folate [139].

In adults with quiescent UC on a 12-week Mediterranean diet pattern, alteration in fecal metabolome prominently involved folate biosynthesis pathways. Higher baseline fiber-degrading Bacteroidia (including *Bacteroides vulgatus*, *B. uniformis*, and *B. acidifaciens*) predicted diet response, suggesting that pre-existing microbiome composition conditions influence the effectiveness of the diet driving folate-linked pathways [140]. Diet-based studies in UC and analyses of IBD with comorbid NAFLD similarly find that responders or high-risk phenotypes are distinguished by microbial community structures tied to folate biosynthesis [124].

##### Gastritis

Folate absorption and utilization depend on gastric acidity and intact mucosa. Studies show folate malabsorption may be disrupted in atrophic gastritis and that folate supplementation might improve gastric atrophy and mucosal repair, supporting a potential mechanistic link between disturbed folate, gastric inflammation, and disease progression [141]. After *H. pylori* eradication, patients who developed or maintained gastric atrophy or intestinal metaplasia had a distinct gastric microbiota with reduced microbial folate biosynthesis [142]. In *H. pylori*-infected transgenic, insulin–gastrin mice, both antibiotics and higher dietary folate reduced helicobacter-induced gastric pathology, but antibiotics depleted menaquinone-producing Bacteroidaceae and altered microbial vitamin K output, leading to severe vitamin K-deficiency anemia despite folate supplementation [143].

##### Small Intestinal Bacteria Overgrowth (SIBO)

SIBO triggers mucosal inflammation, alters epithelial permeability, and interferes with nutrient absorption and host metabolism. Patients with methane-predominant SIBO had elevated serum folic acid despite low fiber and lactose intake, whereas other SIBO subtypes showed different nutrient deficiency patterns [144]. In a larger dyspeptic cohort, patients with SIBO were more likely to have elevated serum folate, consistent with bacterial folate production in the small intestine contributing to systemic levels [145]. However, metagenomic pathway analysis in SIBO showed that predicted one-carbon pool by folate was significantly reduced. This may indicate that despite higher host folate, the community’s folate-linked metabolic capacity is disrupted with specific *saccharolytic* genera (e.g., *Coprococcus_2*, *Butyrivibrio*) potentially contributing to both gas-related symptoms and altered folate metabolism [146].

##### Irritable Bowel Syndrome (IBS)

A rodent study found that IBS was associated with increased alpha diversity and Clostridial abundance along with a decrease in blood folate [147].

##### Gastrointestinal Summary

The core mechanism of GI disorders may involve the loss of folate-producing commensals and dysregulated microbial folate biosynthesis, compromising mucosal renewal, barrier integrity, and immune homeostasis. Altered folate-related microbial pathways has been linked to inflammation across IBD, gastritis, SIBO, and IBS, but contradictions remain regarding the direction of folate change. For example, elevated folic acid in methane-predominant SIBO and post *Helicobacter pylori* gastritis contrasts with the lower folate observed in active IBD and IBS models. Human evidence is largely observational and therefore low certainty, while animal and mechanistic data remain very low certainty because of indirectness and limited translational precision (see Supplementary Table S2.5). Future work should prioritize prospective preclinical-to-clinical cohorts, mucosal (rather than fecal) sampling to localize folate-producing taxa, and randomized trials testing whether targeted folate or microbial restoration alters disease course. Risk of bias was fair to good (NOS) for the human observational studies—including one good-quality prospective cohort—some concerns (RoB 2) for the one human dietary RCT, and moderate (SYRCLE) for the supporting animal studies (Supplementary Table S2.5).

#### 3.3.3. Cancer

The carcinogenic consequences of abnormal folate metabolism arise from the convergence of genomic instability and disrupted methylation homeostasis. Folate deficiency destabilizes the genome through uracil misincorporation and compromised DNA repair, while simultaneously creating a permissive epigenetic landscape via global DNA hypomethylation activating proto-oncogenes and locus-specific promoter hypermethylation silencing tumor suppressor genes. The relationship between folate and cancer risk is nonlinear: deficiency promotes tumor initiation, whereas excess folic acid may paradoxically accelerate progression of pre-existing neoplasms [148].

Using host-microbiota co-metabolic network models, folate metabolism was found to be one of 17 key co-metabolites that primarily influence microbial function in colorectal cancer (CRC)-associated bacterial communities [149].

Liss et al. identified enrichments of *Bacteriodes* and *Streptococcus* species in the human fecal microbiome of individuals with prostate cancer with bacterial-associated folate metabolism enriched in non-cancer patients [150]. Wakamori et al. found that *Odoribacter* and *Desulfovibrio* as well as folate metabolism were associated with prostate cancer in both mice and humans [151].

Treatment-naive acute lymphoblastic leukemia children demonstrated a dysbiotic microbiome enriched in *Enterococcus faecium*, oral commensals such as *Rothia dentocariosa*, multiple opportunistic species and a reduction in *Anaerostipes hadrus* and *Intestinibacter bartlettii*. Their microbiome relied more on protein and amino acid catabolism, while the microbiome of non-cancer patients possessed enhanced pathways for carbohydrate and folate metabolism [152]. In newly diagnosed extranodal natural killer/T cell lymphoma patients, the microbiome showed Enterobacteriaceae-dominated dysbiosis and a concomitant decrease in folate biosynthesis pathways [153].

In PTEN-mutation–positive PTEN hamartoma tumor syndrome patients, individuals with cancer showed a distinct microbial composition with an increase in Rikenellaceae and unclassified Clostridia, a decrease in Peptostreptococcaceae, Enterobacteriaceae, and Bifidobacteriaceae, along with enrichment of microbial folate biosynthesis pathways [154].

Folate–microbiome dysregulation in cancer converges on genomic instability and aberrant methylation, with depletion of folate-producing commensals and altered microbial folate biosynthesis intersecting with host uracil misincorporation and locus-specific epigenetic drift to permit tumor initiation and progression. Folate-related microbial signatures are consistent across colorectal, prostate, hematologic, and PTEN-associated malignancies, but they conflict on the direction of microbial folate biosynthesis. For example, folate biosynthesis is enriched in some prostate and PTEN-associated cancers yet depleted in leukemia and lymphoma, suggesting the well-recognized nonlinear, dose- and timing-dependent relationship between folate and carcinogenesis. These studies therefore provide low-certainty evidence for associations between folate-related microbiome alterations and cancer phenotypes, while supporting animal, computational, and mechanistic data remain very low certainty (see Supplementary Table S2.6). Priority research directions include longitudinal pre-diagnostic cohorts, integrated metagenomic–metabolomic profiling and mechanistic studies clarifying when microbial folate is protective versus tumor-promoting in specific tissue contexts. Risk of bias was fair (NOS) across the case-control and cross-sectional human studies, with the principal limitations being modest sample sizes, treatment-exposure confounding, and reliance on inferred microbial folate function (Supplementary Table S2.6).

#### 3.3.4. Psychiatric Disease

Psychiatric disorders are driven by folate-dependent impairment of monoamine neurotransmitter synthesis (via BH_4_ regeneration) and disrupted SAM-dependent methylation of neurodevelopmental gene programs. Homocysteine neurotoxicity through NMDA receptor overstimulation compounds these effects [72,73,74,75,76,77].

One study found that autism spectrum disorder (ASD) patients exhibited a significantly higher abundance of Actinomycetota and a lower abundance of Bacillota on the phylum level and higher abundance of *Bifidobacteriaceae* and *Ruminococcaceae* on the family level but no difference in serum folate levels [155].

Schizophrenia patients showed a reduced relative abundance of Bacillota and increase in Bacteroidota, Actinomycetota, Verrucomicrobia and Synergistetes at the phylum level. At the genus level, there was a relative reduction in *Gemmiger*, *Roseburia*, *Faecalibacterium*, *Coprococcus*, *Fusicatenibacter* and *Butyricicoccus*, while 26 other functional genera (including *Prevotella*, *Akkermansia*, *Streptococcus* and *Romboutsia*) were increased, along with a higher activity of the microbiota folate biosynthesis pathways [156]. Drug-free first-episode schizophrenia patients showed a significant relative reduction in *Bifidobacteria* and *Bacteroides*, along with a decrease in serum folate. Serum folate was positively correlated with relative abundance of *Bifidobacteria* and an analysis found that the folate × *Bifidobacteria* interaction was correlated with general psychopathology scores [157].

Treatment-naive children and adolescents with obsessive–compulsive disorder were found to have a decrease in alpha-diversity with associated folate-related microbial metabolic disturbances [158].

The folate–microbiome axis in psychiatric disease appears dysregulated through reduced microbial folate availability and shifts in folate-producing taxa that compromise BH_4_-dependent monoamine synthesis, SAM-dependent neurodevelopmental methylation, and homocysteine-mediated NMDA excitotoxicity. ASD, schizophrenia, and obsessive–compulsive disorder all demonstrate links between folate-related microbial alterations and symptom-relevant community structure, but contradictions are notable. For example, some schizophrenia cohorts show increased microbial folate biosynthesis while drug-naive first-episode patients show reduced serum folate and *Bifidobacteria*, and ASD patients can exhibit microbiome changes without parallel serum folate differences. Because these data are predominantly cross-sectional and modest in size, the human evidence is low certainty, with mechanistic support remaining very low certainty (see Supplementary Table S2.7). Future research should prioritize longitudinal pediatric and prodromal cohorts, paired stool–serum–CSF folate-form profiling, and interventional studies testing whether modulating folate-producing taxa changes symptom trajectories. Risk of bias was fair (NOS) across the human case-control studies, with the principal limitations being antipsychotic and dietary confounding, modest sample sizes, and absence of treatment-naive replication (Supplementary Table S2.7).

#### 3.3.5. Cardiovascular Disease (CVD)

Cardiovascular disease is driven primarily by hyperhomocysteinemia, which impairs endothelial NO signaling, augments vascular oxidative stress, drives platelet hyperactivation, and promotes LDL oxidation and vascular smooth muscle proliferation [78,79,81]. Atherosclerosis metagenomics in Swedish and Chinese cohorts showed that controls were enriched for *Bacteroides xylanisolvens*, *Roseburia* and *Eubacterium* species that contribute to folate transformation pathways; these taxa and folate-related functions were depleted in patients, nominating folate-producing commensals as candidate anti-atherogenic probiotics [159]. In a case-control study of retinal vein occlusion patients, those with retinal vein occlusion had dysbiosis with a higher Bacillota/Bacteroidota ratio and enriched *Escherichia* and *Shigella*, with a functional prediction indicating reduced microbial folate biosynthesis [160]. In HIV-infected populations, functional inference from large 16S cohorts revealed broad downregulation of microbial thiamine and folate biosynthesis genes that persisted despite antiretroviral therapy, consistent with clinically observed B-vitamin deficiencies and their links to inflammation, CVD, and neurocognitive disease [161].

Depletion of folate-producing and folate-transforming commensals contributes to cardiovascular disease by limiting microbial support for host remethylation of homocysteine, thereby amplifying endothelial dysfunction, oxidative stress, and atherogenesis. Reduced microbial folate biosynthesis potential is consistently found across atherosclerosis, retinal vein occlusion, and HIV-associated vascular risk, but evidence is limited by reliance on inferred function from 16S data rather than measured microbial folate output, and by sparse direct linkage to clinical cardiovascular endpoints. These studies are observational and largely based on inferred functional capacity, yielding low-certainty human evidence and very low-certainty mechanistic support (see Supplementary Table S2.8). Priority research directions include shotgun metagenomic and metabolomic studies measuring microbial folate species in atherosclerosis cohorts, mechanistic models testing whether restoring folate-producing taxa lowers homocysteine and vascular injury, and trials of folate-targeted probiotics as adjuncts to standard cardiovascular care. Risk of bias was fair to good (NOS) across these observational studies, with the principal limitation being heavy reliance on inferred microbial folate function from 16S data rather than measured folate output (Supplementary Table S2.8).

#### 3.3.6. Neurologic Disease

Neurological diseases reflect CNS dependence on 5-MTHF for myelin synthesis, neurotransmitter production, and neuronal proliferation. Deficiency impairs BH_4_ regeneration and myelin methylation, while homocysteine-mediated NMDA overstimulation and mitochondrial dysfunction from folate-dependent mtDNA damage compound neurodegeneration [72,73,74,75,76,77,85,86,87].

In an Alzheimer’s-like, high-fat rat model, combined folate and B12 deficiency worsens insulin resistance, neuroinflammation, and memory, and uniquely perturbs the gut microbiome [162]. Using a Mendelian randomization analysis, *Bifidobacterium* and *Lachnospiraceae* NK4A136 was found to mediate the relationship between B-vitamin (including folate-containing complexes) and Alzheimer’s disease risk [163].

In Parkinson’s disease, metagenomics plus serum metabolomics and community-scale modeling show that microbiota have a predicted reduced capacity to synthesize or supply folate to the host, contributing to the characteristic folate [164].

In elderly individuals, higher folate intake was associated with greater gut microbial beta diversity and distinct taxa linked to better sleep scores [165].

Microbial folate supply to a CNS is critically for myelin synthesis, BH_4_-mediated neurotransmitter production, and mitochondrial DNA integrity, Reduced supply results in downstream homocysteine neurotoxicity and impaired methylation, which compounds neurodegeneration processes. Such findings are consistent in Alzheimer’s disease, Parkinson’s disease, and aging-related sleep phenotypes, but they diverge across the taxa implicated. For example, *Bifidobacterium* and *Lachnospiraceae* NK4A136 are depleted in Alzheimer’s disease while there is broader community-scale depletion in Parkinson’s disease. Human observational data therefore provide low-certainty evidence, while animal and modeling studies remain very low certainty (see Supplementary Table S2.9). Future research should prioritize longitudinal aging cohorts with paired neuroimaging, multi-omic measurement of microbial folate production, and interventional trials assessing whether targeted folate or microbial modulation slows cognitive or motor decline. Risk of bias was fair to good (NOS) across the human observational studies—including one good-quality Mendelian randomization analysis—and moderate (SYRCLE) for the supporting animal data, with the principal limitation being inferred rather than measured microbial folate flux (Supplementary Table S2.9).

#### 3.3.7. Immune Disorders

Immune disorders are linked to folate through impaired T-regulatory lymphocyte maintenance, compromised hematopoiesis from deficient nucleotide synthesis, and methylation-driven dysregulation of cytokine expression and immune cell differentiation [69,70,71,83].

Different profiles of microbiome-derived B-vitamins are associated with guttate versus plaque psoriasis, with folate enriched in plaque psoriasis relative to guttate disease [166]. The mechanism by which dysbiosis may be related to increased inflammatory disease risk could involve shifts away from folate-producing communities [167].

In septic compared to non-septic critical patients admitted to the intensive care unit during the same period, the septic patients showed a correlation between serum folate concentrations and *Sellimonas* [168].

A longitudinal study of 63 infants from 3 weeks to 12 months of life demonstrated that early enrichment of *E. coli* and *Klebsiella pneumoniae* and depletion of *B. fragilis* was linked to reduced expression of microbial folate biosynthesis pathways [169].

A cross-sectional inpatient study compared 16S rRNA profiling of the stools from children at “high risk” (cancer, transplant, sickle cell disease) and low risk of *Clostridioides difficile* infection. In the sickle cell subgroup, folic acid supplementation was associated with higher gut microbial alpha diversity, suggesting that folate status may beneficially modulate microbiome richness in this high-risk pediatric population [170].

Folate–microbiome dysregulation in immune disorders reflects shifts away from folate-producing commensals that constrain T-regulatory maintenance, methylation-dependent cytokine programming, and nucleotide-dependent hematopoietic responses, resulting in a predisposition to inflammatory and infection-related phenotypes. Evidence is consistent across psoriasis, sepsis, infant immune programming, and pediatric high-risk populations in linking folate-related microbial alterations to immune outcomes, but findings diverge in direction. For example, plaque psoriasis is enriched in microbial folate while infants with dysbiotic *E. coli* and *Klebsiella* expansion show depleted folate biosynthesis. This may indicate that the immunological consequences of microbial folate depend strongly on host context. The evidence is low certainty for human associations and very low certainty for mechanistic support (see Supplementary Table S2.10). Priority research directions include early life longitudinal cohorts, mechanistic studies of folate-mediated immune cell programming, and trials testing whether folate-producing probiotics modulate inflammatory or infectious disease risk. Risk of bias was fair to good (NOS) across these observational studies, including one good-quality prospective infant cohort, with the principal limitations being heterogeneous clinical settings and incomplete adjustment for medication and dietary exposures (Supplementary Table S2.10).

#### 3.3.8. Female Reproduction

Adverse reproductive outcomes reflect the critical dependence of embryogenesis on folate-dependent OCM. Deficient nucleotide synthesis underlies failed neural tube closure, hyperhomocysteinemia impairs placental vascular development, and both deficiency and excess may perturb the precise methylation patterns required for normal embryonic development.

A longitudinal study of rhesus macaques found that pregnancy microbiomes were enriched for butyrate-production pathways, while the postpartum microbiome showed increased abundance of pathways involved in folate transformation [171]. In a rat pregnancy model, maternal folate status was positively associated with *Lactobacillus*- and *Romboutsia*-related taxa [118].

Preeclampsia was associated with significantly lower red blood cell total folate, 5-MTHF, and 5,10-CH_2_-THF, alongside markedly reduced gut microbiota alpha-diversity and altered community composition across multiple taxonomic levels. Alpha-diversity indices correlated positively with red blood cell (RBC) folate forms [172]. In early-onset preeclampsia, enrichment of *Blautia*, *Pauljensenia*, *Ruminococcus*, and *Collinsella* species and a reduction in *Bacteroides* and *Phocaeicola* species were found along with a lack of a folate-enriched functional profile [173].

It is believed that the core mechanism in adverse reproductive outcomes is a deficit of folate-dependent nucleotide synthesis and methylation during embryogenesis and placentation, with reduced microbial folate supply and dysbiosis-driven hyperhomocysteinemia compounding host insufficiency to impair neural tube closure, placental vascular development, and epigenetic programming. Lower folate status and depleted folate-related microbial signatures are consistently linked to adverse outcomes in pregnancy and preeclampsia, but the evidence remains observational, and contradictions persist regarding which specific taxa drive folate supply across pregnancy stages. Human evidence remains observational and therefore low certainty, while animal and mechanistic evidence is very low certainty despite offering coherent biological support (see Supplementary Table S2.11). Future research should prioritize prospective preconception-to-postpartum cohorts with serial microbiome and folate-form measurements, mechanistic placental and embryonic studies, and randomized trials testing whether folate-producing probiotics complement standard supplementation in reducing preeclampsia or neural tube defect risk. Risk of bias was fair to good (NOS) across the human case-control studies and moderate (SYRCLE) for the supporting animal studies, with the principal limitations being modest sample sizes and observational designs that cannot establish temporal precedence across pregnancy stages (Supplementary Table S2.11).

### 3.4. Interventions Which Change the Folate–Gut Axis in Non-Disease States

Evidence quality for each intervention category was assessed using the OCEBM 2011 framework (Table 2).

#### 3.4.1. Probiotic Interventions

Probiotic intervention studies in vivo show that folate-producing probiotics can correct folate deficiency and remodel dysbiosis beyond the effect of isolated folic acid supplementation. In a folate-deficient rat model, *L. plantarum* GSLP-7 restored serum folate while reversing gut dysbacteriosis [174].

*L. reuteri* 6475 was found to suppress the production of the proinflammatory cytokine tumor necrosis factor (TNF) through production of 5,10-methenyl-THF polyglutamates, directly demonstrating the anti-inflammatory effect of folate [175].

Direct administration of *B. thetaiotaomicron* ameliorated high fat diet (HFD)-induced steatosis and raised gut–liver folate levels [176]. A combination probiotic with *Bacteroides xylanisolvens* and *Clostridium butyricum*, which reciprocally exchange of folate and pABA, outperformed single-strain probiotics in improving metabolic syndrome in HFD mice [177]. Seed-derived *Phytobacter* sp. RSE02 carries genomic clusters for folate, biotin, and B12 synthesis and lowers cholesterol and adiposity in HFD mice [178].

Probiotic treatment during pregnancy can provide vertical programming of microbiome folate production. In pregnant women, a *Bifidobacterium*–*Lactobacillus*–*Streptococcus* probiotic increased folate biosynthesis in maternal microbiota and enhanced folate biosynthesis in early meconium [179]. Maternal probiotics also increased *Bifidobacterium* and *Bacteroides thetaiotaomicron* in infants and upregulated folate pathways [180]. In a mouse model of preeclampsia, *Akkermansia muciniphila* reversed fecal metabolic disturbances in pathways that include folate metabolism [181].

In children with ASD, microbiota transfer therapy increased the abundance of many fiber-consuming and beneficial microbes which were reduced at baseline, namely *Prevotella* (*P. dentalis*, *P. enoeca*, *P. oris*, *P. meloninogenica*), *Bifidobacterium bifidum*, and *Desulfovibrio piger*. Microbiota transfer therapy also enhanced microbiota genes encoding enzymes involved in folate biosynthesis [182].

In the RISTOMED trial in older adults, adding the multi-strain probiotic VSL#3 to an anti-inflammatory diet increased fecal *Bifidobacteria* and significantly raised plasma folate [183], while probiotic-fermented milk in a preeclampsia–hypertension model modifies gut communities and one-carbon/folate-linked metabolic pathways in parallel with blood pressure reduction [184].

Wild-type *L. reuteri* inactivated folC genes but did not influence folate transporter expression [175]. In a canine crossover trial, a diet with *Enterococcus faecium* did not change folate [185].

Probiotic studies span Levels 2–4 for human trials and crossover studies at Level 2–3 with animal model studies rated as Level 3, resulting in an overall grade of B.

#### 3.4.2. Prebiotic Interventions

In human trials, inulin or inulin-type fructans selectively enrich *Bifidobacterium*, *Lactobacillus*, and other saccharolytic taxa and are repeatedly associated with upregulated predicted pathways for folate biosynthesis, coinciding with improvements in glycemic control, adiposity, or MASLD indices [186,187,188,189,190,191]. Kefiran, a kefir polysaccharide, remodeled HFD-induced dysbiosis, increasing *Parabacteroides* and *Alistipes*, and enhancing hepatic folate pathways [192]. In animal models, Ganoderma meroterpene derivatives enrich folate-producing *Bacteroides* (*B. xylanisolvens*, *B. thetaiotaomicron*, *B. dorei*, *B. uniformis*) [193]. In T2D mouse models, a rapeseed meal polyphenol extract modulated microbial folate pathways and improved insulin sensitivity [132]. Similarly, a *Sanghuangporus vaninii* mixture modulated folate biosynthesis and improved metabolic indices [133].

In dextran sulfate sodium-induced colitis, tannic acid, a bioactive polyphenol found in various phytogenic foods and medicinal plants, reshaped the gut microbiota by enriching *Prevotella*, *Eubacterium siraeum*, and *Enterorhabdussiraeum*, with this microbial shift associated with increased colonic folate [194]. In a mouse model of subacute colitis, aloe polysaccharides increased SCFA-producing genera (e.g., *Akkermansia*, *Blautia*), alleviated inflammation and barrier dysfunction, and upregulated 6-pyruvoyltetrahydropterin synthase within the folate biosynthesis pathway [195].

In an ex vivo study, 3’SL human milk oligosaccharide modulated community structure in an age-dependent, *Bifidobacteria*-driven manner, with folic acid being uniquely increased in adults [196].

In Holstein calves, early-life galacto-oligosaccharide supplementation reshaped hindgut microbial functions with altered fecal and serum folate [197].

In neonatal rats, early dietary nucleotides, a major nitrogen-containing substance in human milk, shifted the gut microbiome toward beneficial taxa (including *Akkermansia* and *Romboutsia*) and altered the predicted capacity for folate synthesis [198].

Prebiotic studies are predominantly Level 3 (animal models) and Level 5 (ex vivo/in vitro), with limited Level 2 human RCT data, resulting in an overall grade of C.

#### 3.4.3. Dietary Interventions

In a mouse pregnancy model, undernourishment and HFD reduced circulating folate and certain lactobacilli taxa while only HFD altered expression of folate transporters in the maternal gut, placenta, and fetal gut [199]. In a rat pregnancy model, an obesogenic cafeteria diet lowered maternal and offspring folate levels and shifted gut microbiota composition with altered maternal–offspring microbiome transmission patterns [118].

In adults with quiescent UC, a 12-week Mediterranean diet prominently altered fecal folate biosynthesis pathways [140]. When nuns switched from refined modern wheat to whole-meal ancient wheat, serum folic acid levels decreased. This ancient-grain diet also enhanced microbial carbohydrate metabolism and volatile organic compound production [200].

Dietary studies include small human crossover trials (Level 2–3) and animal models (Level 3), resulting in an overall grade of C.

#### 3.4.4. Folate Intervention

Exogenous folic acid or folate-enriched foods consistently modulate community structure and function, but with context-specific signatures. Poultry, broilers and laying hens supplemented with folic acid show shifts in the Bacillota/ Bacteroidota balance, enrichment of saccharolytic/SCFA-producing taxa (e.g., *Ruminococcus*, Lachnospiraceae NK4A136, *Akkermansia*) and reduced pathogenic or inflammatory genera, alongside increased acetate, propionate, or butyrate [201,202,203,204,205]. Rumen-protected folate [206,207] or folate combined with branched-chain volatile fatty acids increases cellulolytic bacteria (*Ruminococcus* spp., *Fibrobacter succinogenes*) and greater microbial protein synthesis [208]. In weaned piglets, dietary folate supplementation increased several *Lactobacillus* species (*L. reuteri*, *L. salivarius*, *L. mucosae*) and SCFA acetic and valeric acid with acetic acid concentrations correlating positively with *Lactobacillus* [16]. Folate-enriched fermented milks produced with high-folate lactic acid partially restored a dysbiotic microbiota more effectively than equivalent synthetic folic acid [209,210]. In vitro human fecal slurry experiments similarly show that folic acid and 5-methyl-THF shift community composition toward *Lactobacillus*, *Bifidobacterium*, and *Pediococcus* and alter SCFA profiles, confirming that folate itself is a selective pressure on the microbiota [211]. In *C. elegans* models, synthetic folic acid primarily benefits the host indirectly by providing pABA-Glu to *E. coli*, boosting bacterial folate synthesis [41].

Maternal folate and inositol supplementation in NTD-prone mice is accompanied by distinct shifts in maternal and offspring microbiota, suggesting that the preventive effects of folate on neurodevelopment are modulated by, and feed back onto, folate-sensitive gut consortia [17,205].

In hyperuricemia, folic acid lowers uric acid and rebalances the microbiota [212,213]. In IBS-like visceral pain, folic acid supplementation reduces H_2_S-producing Clostridiales [147].

Direct folic acid supplementation in HFD mice suppresses weight gain in conventional but not in germ-free animals, confirming the microbiome dependence of folate’s anti-obesity effects [214,215]. Maternal folic acid supplementation protects offspring from MASLD by shifting offspring microbiota and dampening hepatic inflammation [216].

In early Alzheimer’s disease, the multinutrient Fortasyn Connect (including B-vitamins) increased *Bifidobacterium* and *Lactobacillus* and enhanced microbiota-mediated B-vitamin handling in the aging gut–brain axis [217].

Collectively, these interventions suggest that optimizing folate status with attention to form (synthetic vs. microbially produced), dose, and microbiome context could allow more precise manipulation of one-carbon metabolism, SCFA production, and mucosal immunity than folate pharmacology alone.

Folate supplementation studies are predominantly Level 3 animal models, Level 5 in vitro data and limited Level 2 human RCTs. Folate-enriched fermented milk studies provide Level 2–3 evidence, resulting in an overall grade of C.

#### 3.4.5. Other Intervention

In a CRC mouse model, curcumin treatment reduced harmful bacteria, including *Ileibacterium*, *Monoglobus* and *Desulfovibrio* and increased the abundance of *Clostridia_UCG-014*, *Bifidobacterium* and *Lactobacillus*. The 7,8-dihydropteroic acid, a folate precursor, was restored by curcumin [218]. Using constraint-based metabolic modelling with in vitro experiments, silver nanoparticle-induced reactive oxygen species-based CRC treatment increased *Enterococcus durans* extracellular folate concentrations [219].

Other intervention studies include one animal model study (Level 3) and one in vitro/computational study (Level 5), resulting in an overall grade of D.

#### 3.4.6. Intervention Evidence Summary

Overall, the 54 intervention studies examining the folate–gut microbiome axis span OCEBM Levels 2–5. Probiotic interventions (*n* = 12) achieved the strongest evidence grade (Grade B), supported by multiple human controlled trials (Level 2), animal model studies (Level 3), and human observational studies (Level 4). Prebiotic interventions (*n* = 15) received Grade C, with evidence primarily from animal models (Level 3), limited human RCTs (Level 2), and ex vivo/in vitro mechanistic studies (Level 5). Dietary interventions (*n* = 4) also received Grade C, based on small human crossover trials (Level 2–3) and animal models (Level 3). Folate supplementation studies (*n* = 21) were graded C, reflecting predominantly animal model evidence (Level 3) and in vitro data (Level 5), and limited Level 2 human RCTs. Other interventions (*n* = 2), comprising one animal model and one computational/in vitro study, received Grade D. No intervention category achieved Grade A. The predominance of animal model studies and the scarcity of adequately powered human RCTs across all categories underscores the critical need for well-designed clinical trials with folate-specific microbiome endpoints to advance the translational potential of these findings. Across all intervention categories, only probiotic interventions achieved Grade B evidence; prebiotic, dietary, and folate supplementation interventions were all rated Grade C or below. No intervention category reached Grade A, highlighting the paucity of well-designed randomized controlled trials in this field.

Translating the intervention literature into GRADE terms, probiotic interventions provide the strongest body of evidence, generally corresponding to low- to moderate-certainty evidence because several human controlled studies are available, although many remain small and use surrogate endpoints (see Supplementary Tables S2.12–S2.16). Prebiotic, dietary, and folate supplementation studies generally provide low-certainty evidence because they rely heavily on animal studies, small human trials, and mechanistic endpoints, whereas other interventions remain very low to low certainty. No intervention category presently reaches high certainty. Risk of bias was low to some concerns (RoB 2) for the small number of human RCTs identified—most often downgraded for open-label design or incompletely reported allocation—moderate to serious (ROBINS-I) for non-randomized human intervention studies, and moderate (SYRCLE) for the predominant animal experimental literature, where allocation concealment and blinded outcome assessment were rarely reported (Supplementary Tables S2.12–S2.16).

## 4. Discussion

This systematic review synthesizes current evidence on folate–gut microbiome interactions, highlighting their relevance to human health across metabolic, gastrointestinal, oncologic, neuropsychiatric, cardiovascular, immunologic, and reproductive disease contexts.

### 4.1. Synthesis Beyond Prior Reviews

Prior reviews of folate and the microbiome have focused largely on cataloguing folate-producing taxa or on supplementation outcomes in single disease contexts. The present synthesis advances the field in three ways. First, by integrating in vitro, in silico, and ecological evidence with disease-specific findings across eight organ systems, it shifts the framing from a list of folate-producing species toward a community-level economy in which prototrophs and auxotrophs are coupled by cross-feeding and in which most microbial folate is retained intracellularly until cell turnover. Second, the inclusion of 54 intervention studies graded by OCEBM and GRADE distinguishes patterns supported by mechanistic and controlled human evidence from those still confined to associative or single-cohort observations, providing a transparent map of where the field is genuinely advancing versus where claims have outrun the data. Third, the disease-by-disease syntheses surface recurring discrepancies, particularly around the directionality of microbial folate change. This is something that prior reviews have generally overlooked and motivates a unified conceptual model rather than a parallel set of organ-specific narratives.

### 4.2. An Integrative Model of the Host–Microbe One-Carbon Network

**Figure 5:**
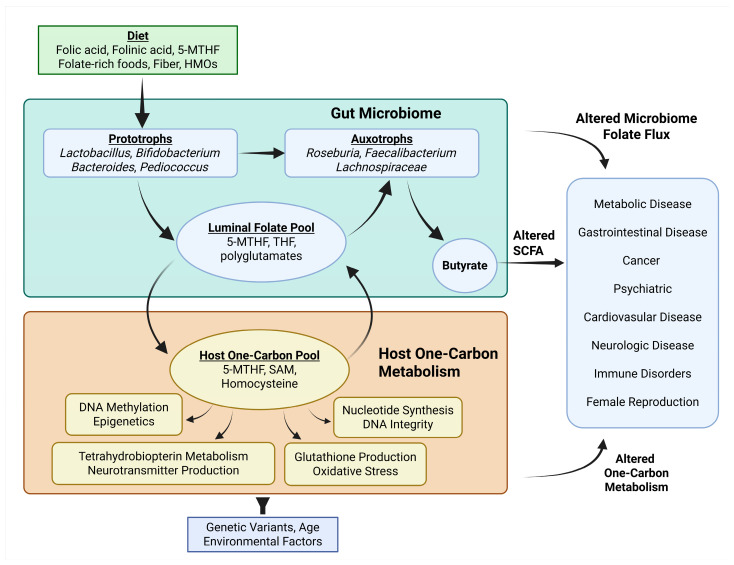
Integrative host–microbe one-carbon network. Folate functions as a shared currency between the microbial layer (prototrophs contribute to a luminal folate pool and are coupled to auxotrophs by cross-feeding) and the host layer (host folate pool feeding DNA methylation, nucleotide synthesis, neurotransmitter production, and glutathione production). Dietary inputs and host (genetic variants, age) and other environmental modifiers shape both compartments. Bidirectional exchange between the luminal and host pools links microbial folate flux to host one-carbon metabolism, and perturbations propagate to disease-relevant outcomes across organ systems. Disease processes feed back on diet, microbial composition, SCFA production and host folate handling producing self-reinforcing loops. Created in BioRender. Frye, R. (2026) https://BioRender.com/0lf1nb6.

Drawing the evidence together, we propose that folate is best understood not as a host nutrient that the microbiota incidentally modify, but as a shared currency of a host–microbe one-carbon network (Figure 5). In this model, dietary inputs (folic acid, reduced folates, fermentable fibers, human milk oligosaccharides) and host modifiers (MTHFR and RFC variants, age, pregnancy state) jointly shape two coupled folate compartments: a microbial layer composed of folate prototrophs and auxotrophs linked by cross-feeding, and a host layer in which absorbed folate fuels SAM-dependent methylation, homocysteine remethylation, and thymidylate synthesis. The luminal folate pool is the interface between these layers, both supplied by microbial release after cell turnover and consumed by host enterocyte uptake, while host folate status reciprocally selects for or against folate-producing taxa. Disease-relevant outcomes, metabolic, gastrointestinal, oncologic, neuropsychiatric, cardiovascular, immune, and reproductive, emerge when this network is perturbed at any node, and they typically feed back on diet, microbial composition, and host folate handling, producing the self-reinforcing loops we observe across conditions.

Three properties of this model help reconcile findings that appear discordant when each disease is considered in isolation. First, the network is non-additive: identical changes in microbial folate biosynthesis can produce opposite host consequences depending on host genotype, life stage, and the local tissue context. For example, the same microbial folate enrichment that supports neurodevelopment in early life may promote colonic neoplasia in genetically susceptible adults. Second, the network is compartmentalized: serum, erythrocyte, mucosal, and luminal folate pools are imperfectly correlated, so a study that measures one compartment cannot be assumed to reflect another. Third, the network is dynamic: longitudinal disease progression remodels both the microbial and host arms, such that early disease states often show preserved or even enriched microbial folate capacity, while later, more inflamed states show progressive depletion. Reading the literature through this framework converts a series of seemingly contradictory observations into a coherent, if more demanding, picture of where, when, and in whom microbial folate matters.

### 4.3. Reconciling Contradictory Evidence on Microbial Folate Synthesis

A striking feature of the literature is that several disease states show ostensibly contradictory findings: studies report both elevated and reduced microbial folate biosynthesis or folate-pathway abundance within the same condition. We highlight the most reproducible discrepancies and propose mechanistic explanations grounded in the integrative model above. These contradictions are summarized in Table 3, which collates the principal disease states in which divergent reports have appeared, the direction of the apparent disagreement, and the most plausible biological or methodological reasons for it.

Several methodological factors recur across these examples and merit explicit recognition. (i) Compartment matters: serum, erythrocyte, fecal, and mucosal folate pools are imperfectly correlated, and inferences drawn from one compartment regularly disagree with those drawn from another. (ii) Inferred function is not measured function: 16S-based predictions of microbial folate biosynthesis (e.g., PICRUSt-derived KEGG modules) overestimate capacity in inflamed or low-diversity communities and cannot distinguish active flux from genomic potential. (iii) Disease stage and treatment alter the microbiome at least as strongly as the underlying disease, so cross-sectional cohorts without stratification by stage, medication, and dietary intake should be interpreted cautiously. (iv) Folate form is biologically consequential: synthetic folic acid, reduced folates, and microbially produced 5-MTHF interact differently with host transporters and microbial communities, and aggregating across forms obscures real biological effects.

Recognizing these sources of apparent contradiction reframes them as informative rather than embarrassing: they identify exactly the network nodes—compartment, life stage, host genotype, treatment exposure, and folate form—at which future studies must measure carefully if microbial folate is to move from a correlate of disease to a tractable therapeutic target. The integrative model in Figure 5 makes these dependencies explicit and provides a scaffold for designing the next generation of mechanistic and clinical studies.

### 4.4. Therapeutic and Translational Perspectives

Folate is involved in many critical disease processes. This involves diet, microbiome health and supplementation with the proper folate at the correct dose. These factors are important in decreasing disease risk and can be used as part of treatment regimens along with standard treatments to possibly improve outcomes. The OCEBM evidence grading of intervention studies demonstrates that while probiotic interventions have achieved the strongest evidence base (Grade B), all other categories remain at Grade C, underscoring the need for adequately powered human randomized controlled trials. What emerges is that many diseases which appear distinct in their etiology may rather share fundamental abnormalities in common pathophysiology which manifests itself differently clinically, depending on other factors. The synthesis of a unifying folate–microbiome axis in which variation in microbial folate production, host folate handling, and convergent mechanisms—including methylation dysregulation, oxidative stress, and immune activation—contribute to a common mechanistic scaffold provides insight into how disorders as disparate as neurodevelopmental and oncological disorders can arise from similar fundamental abnormalities. The genetic evidence from *folP* knockout studies and germ-free mouse experiments provides some of the strongest causal evidence to date that microbial folate production is not merely a biomarker but a functional driver of host metabolic and disease phenotypes.

### 4.5. Current Knowledge Gaps and Future Research Needs

#### 4.5.1. Limitations of Current Evidence

From a GRADE perspective, the certainty of evidence for clinically meaningful folate–microbiome effects remain limited despite the breadth of mechanistic and translational studies identified in this review. Most human evidence is observational or derived from small interventional studies and therefore contributes low-certainty evidence for associations between microbial folate pathways and metabolic, gastrointestinal, oncologic, neuropsychiatric, cardiovascular, immunologic, and reproductive phenotypes. The substantial in vitro, animal, ex vivo, and in silico literature provide coherent biologic support, but because these studies are indirect, they remain very low certainty for human clinical inference. Only a subset of probiotic and diet-related intervention studies approaches moderate certainty, and no domain reaches high certainty, underscoring the need for adequately powered randomized trials with prespecified folate-related microbiome endpoints, direct measurement of folate forms, and standardized intervention formulations. In addition, current evidence is primarily derived from associational and observational studies and is insufficient to establish direct causal relationships; well-designed randomized controlled trials in human populations are needed to verify these associations.

A parallel risk-of-bias appraisal using design-appropriate tools (RoB 2, ROBINS-I, Newcastle-Ottawa Scale, and SYRCLE for clinical and animal studies, with a structured narrative appraisal for mechanistic work; Supplementary Table S2) reinforces this picture. The small number of human randomized controlled trials identified were judged as low risk or some concerns on RoB 2—most often downgraded for open-label intervention delivery or incompletely reported allocation concealment—and the broader human observational literature was largely judged fair on the Newcastle-Ottawa Scale, with principal weaknesses in dietary intake adjustment, inferred-function analyses, and limited control of medication and stage-of-disease confounders. The non-randomized human intervention studies clustered at moderate ROBINS-I risk, with serious risk in open-label before–after designs lacking concurrent controls, and the dominant animal experimental literature was uniformly moderate on SYRCLE, primarily because allocation concealment and blinded outcome assessment are rarely reported in this field. The convergence of bias appraisal with the GRADE certainty assessment indicates that the limitations are not concentrated in individual studies but are structural to the evidence base, and they delineate the methodological priorities—pre-registered, allocation-concealed, outcome-blinded human trials with measured rather than inferred microbial folate output—that must be addressed for the certainty of evidence to advance.

There is limited research investigating the species resident in the human GI tract capable of microbial folate synthesis. Related to this, how certain factors may affect the absorption of bacterially synthesized folate is not fully understood. Additionally, although folate status appears to influence the enteric microbiome, questions remain about the functional implications of these changes, as well as whether different forms of folate can have distinct effects on the microbiome. The latter point becomes relevant in the context of diseases in which dysbiosis contributes to pathophysiology.

The core research gaps include investigating the quantitative contribution of microbially synthesized folate to total folate pools in humans across life stages and disease states, the regulatory factors (diet, SCFAs, pH, host genetics) that control folate biosynthetic activity in vivo, and the mechanistic links between specific folate-producing or folate-dependent taxa and disease outcomes. Other secondary gaps include investigating the differential effects of folic acid vs. reduced folates vs. probiotic-derived folate on microbiome structure and function, the long-term safety of manipulating microbial folate production, and the optimal combinations of diet, prebiotics, and probiotics to sustain folate-producing communities.

#### 4.5.2. Priority Research Areas

Future in vivo studies are needed in humans to understand the effects of the microbiome on folate status and how it contributes to disease. Stable isotope tracing (e.g., labeled 5-formyl-THF delivered to the colon) combined with metabolomics and compartment-specific sampling could directly quantify absorption of microbially produced folate and its incorporation into systemic pools. Multi-center human cohorts with standardized dietary, genetic, and microbiome assessments would enable robust evaluation of how folate metabolism gene polymorphisms (e.g., MTHFR, DHFR, RFC variants) interact with enteric microbiota in relevant disorders. Integrative ‘omics’ approaches, which include metagenomics, metatranscriptomics, and host epigenetic profiling, may clarify how perturbations in microbial folate synthesis affect OCM and gene regulation.

In addition, although the evidence poses probiotics as a safe and promising way of delivering folate, especially in disorders where folate metabolism and gut dysbiosis accompany each other, future clinical trials are needed to solidify the safety and efficacy of such treatment.

## 5. Conclusions

In this review, we explored how folate status and the enteric microbiome are heavily interlinked. We have highlighted the bidirectional nature of this relationship, suggesting that changes to the microbiome will most likely be accompanied by changes to folate status and vice versa. Alterations in this folate–microbiome axis were consistently associated with a wide range of metabolic, GI, cardiovascular, neurologic, psychiatric, immune, and reproductive disorders, although causal relationships remain incompletely defined.

This review found three core findings: First, a subset of gut bacteria, particularly *Bifidobacterium* and selected lactic acid bacteria, constitute a core folate-producing guild whose activity is shaped by cross-feeding with auxotrophic microbes and the colonic microenvironment. Second, disruptions in this folate–microbiome axis may contribute to disease through impaired nucleotide synthesis, altered methylation, and immune dysregulation. Third, therapeutic strategies that combine dietary folate optimization, targeted use of folate-producing probiotics, and consideration of host genetics offer promising avenues but require rigorous clinical trials to define efficacy and safety. Priority clinical targets include identifying individuals with concurrent folate pathway polymorphisms and dysbiosis, restoring folate-producing taxa in high-risk groups, and personalizing folate form while maximizing biologically active folate delivery.

## Figures and Tables

**Table 1:** Disease states associated with abnormal folate metabolism and the pathological folate mechanisms driving each disease category.

Primary Pathological Folate Mechanism(s)	Key Downstream Consequences
**Metabolic Disease**	
Disrupted methylation homeostasis Impaired mitochondrial one-carbon flux Hyperhomocysteinemia	Epigenetic dysregulation of metabolic genes Impaired lipid/glucose homeostasis Insulin resistance Oxidative stress
**Gastrointestinal Disorders**	
Impaired nucleotide synthesis Disrupted methylation homeostasis Genomic instability	Compromised mucosal renewal and repair Aberrant immune activation Barrier dysfunction Increased neoplastic risk
**Cancer**	
Genomic instability Disrupted methylation Carcinogenesis	Proto-oncogene activation Tumor suppressor silencing Elevated somatic mutation burden Impaired immune surveillance
**Psychiatric Disease**	
Impaired neurotransmitter synthesis Disrupted methylation of developmental genes Hyperhomocysteinemia	Reduced monoamine neurotransmitters Impaired synaptic plasticity/myelination NMDA-mediated neurotoxicity
**Cardiovascular Disease**	
Hyperhomocysteinemia Disrupted vascular methylation Cardiovascular/thrombotic consequences	Endothelial dysfunction LDL oxidation Platelet hyperactivation Prothrombotic state
**Neurologic Disease**	
Impaired CNS folate transport Disrupted methylation Mitochondrial dysfunction	Impaired myelination Reduced neurotransmitter synthesis Homocysteine neurotoxicity mtDNA damage
**Immune Disorders**	
Impaired immune cell proliferation Disrupted T-regulatory cell maintenance Hematologic consequences	Immune dysregulation Proinflammatory state Compromised tolerance Impaired hematopoiesis
**Female Reproduction**	
Impaired embryonic nucleotide synthesis Disrupted developmental methylation Hyperhomocysteinemia	Failed neural tube closure Disrupted placental vascular development Abnormal gene imprinting

**Table 2:** Oxford Centre for Evidence-Based Medicine (OCEBM) 2011 Levels of Evidence and Grades of Recommendation for intervention studies examining the folate–gut microbiome axis.

Intervention Category	Studies	Study Designs (OCEBM Levels)	Overall Grade
Probiotics	12	Level 2: Human RCTs and crossover trials; Level 3: Animal model controlled studies; Level 4: Human observational/before–after studies	Grade B
Prebiotics	15	Level 2: Limited human RCTs (small samples); Level 3: Animal model controlled studies; Level 5: Ex vivo/in vitro mechanistic studies	Grade C
Dietary	4	Level 2–3: Small human crossover/parallel-arm trials; Level 3: Animal model studies	Grade C
Folate	21	Level 2: Limited human RCTs; Level 3: Animal model controlled studies; Level 4: Human observational/cross-sectional studies; Level 5: In vitro fecal slurry experiments	Grade C
Other	2	Level 3: Animal model studies; Level 5: In vitro/computational modeling	Grade D

**Table 3:** Disease states with contradictory evidence on microbial folate biosynthesis and proposed explanations.

Direction of Disagreement	Proposed Explanation
**Obesity**	
Low serum folate yet elevated red blood cell folate; reduced microbial biosynthesis in some cohorts but enriched folate-producing taxa in others.	Compartment dissociation: long-lived erythrocyte folate reflects historical high folic acid intake, while serum and luminal pools reflect current microbial supply. Inflammation-driven changes in folate-binding proteins and unmetabolized folic acid can further decouple measured pools from functional one-carbon flux.
**MASLD/metabolic syndrome**	
Locally enriched duodenal *Lactobacillus*-derived folate alongside reduced overall fecal folate biosynthesis.	Spatial heterogeneity: small-bowel folate-producing communities differ from colonic communities, and fecal sampling underestimates upper-GI prototroph activity. Stage of disease also matters—early metabolic dysfunction can show preserved or enriched microbial folate that progressively declines as fibrosis develops.
**Small intestinal bacterial overgrowth (SIBO)**	
Elevated systemic folate yet reduced predicted folate biosynthesis pathways.	Shift from physiologic colonic biosynthesis to small-intestinal overgrowth places folate-producing organisms where folate is more readily absorbed, raising serum folate even as community-level biosynthetic capacity falls. Methane-predominant subtypes show particularly high folate, consistent with archaeal–bacterial cross-feeding.
**Inflammatory bowel disease**	
Reduced microbial folate biosynthesis in active disease but variable findings in remission and pediatric IBD.	Mucosal inflammation disproportionately depletes oxygen-sensitive folate-producing anaerobes (e.g., *Faecalibacterium*, *Roseburia*); 16S-inferred function overestimates folate capacity when these taxa partially recover in remission without restoring full pathway flux.
**Colorectal and prostate cancer**	
Microbial folate biosynthesis enriched in some prostate and PTEN-associated cancers but depleted in CRC and hematologic malignancies.	Tissue context determines whether microbial folate is protective or tumor-promoting. Folate fuels nucleotide synthesis required for both repair and tumor proliferation; the dose- and timing-dependent effect of folate on carcinogenesis (well-established for host folate) extends to microbial folate and is further modified by mucosal versus systemic exposure.
**Schizophrenia**	
Some cohorts show increased microbial folate biosynthesis while drug-naive first-episode patients show reduced serum folate and *Bifidobacteria*.	Antipsychotic exposure, dietary change, and treatment-related metabolic shifts in chronic patients raise microbial folate capacity, masking the reduced microbial folate that characterizes earlier, untreated disease. Cross-sectional sampling without treatment stratification produces apparent contradictions.
**Autism spectrum disorder**	
Microbiome alterations without consistently parallel changes in serum or RBC folate.	Functional folate insufficiency in ASD is often mediated by folate receptor autoantibodies and cerebral folate transport defects rather than by absolute systemic deficiency, so blood folate may appear normal while CNS and microbial folate flux are altered. Stool-only sampling also misses small-bowel prototroph activity.
**Psoriasis and immune disorders**	
Microbial folate enriched in plaque psoriasis but depleted in dysbiotic infants with *E. coli*/*Klebsiella* expansion.	Immune phenotype depends on the producer identity, not just folate amount: pro-inflammatory taxa carrying folate pathways differ functionally from commensal folate producers, so enrichment of folate biosynthesis can be either beneficial or harmful depending on the carrier organism and host immune state.
**Cardiovascular and aging contexts**	
Inferred microbial folate biosynthesis can appear preserved or even increased in frail older adults despite reduced diversity.	Reliance on 16S-inferred function (e.g., PICRUSt) rather than measured microbial folate output overestimates capacity when folate-pathway-bearing taxa expand at the expense of more diverse, lower-abundance prototrophs. Age-related shifts in microbial composition may reflect compensatory rather than physiologic enrichment.

## Data Availability

No new data were created or analyzed in this study. Data sharing is not applicable to this article.

## Supplementary Materials

The following supporting information can be downloaded at https://www.mdpi.com/article/10.3390/ijms27115048/s1. Reference [220] is cited in the supplementary materials.

## Abbreviations

The following abbreviations are used in this manuscript:

5-MTHF	5-methyltetrahydrofolate
5,10-CH_2_-THF	5,10-methylene-tetrahydrofolate
10-formyl-THF	10-formyl-tetrahydrofolate
ASD	Autism spectrum disorder
BCAA	Branched-chain amino acids
BH_4_	Tetrahydrobiopterin
CP	Chronic pancreatitis
CRC	Colorectal cancer
CVD	Cardiovascular disease
DHF	Dihydrofolate
DHFR	Dihydrofolate reductase
DHPPP	6-hydroxymethyl-7,8-dihydropterin pyrophosphate
DNA	Deoxyribonucleic acid
dTMP	Deoxythymidine monophosphate
dUMP	Deoxyuridine monophosphate
FFM	Free folate medium
FPGS	Folylpoly-γ-glutamate synthetase
GGH	Glutamate carboxypeptidase II
GI	Gastrointestinal
GTP	Guanosine triphosphate
HFD	High-fat diet
HIV	Human immunodeficiency viruses
IBD	Inflammatory bowel disease
KEGG	Kyoto Encyclopedia of Genes and Genomes
L-DOPA	Levodopa (L-3,4-dihydroxyphenylalanine)
LC-MS	Liquid chromatography–mass spectrometry
LPS	Lipopolysaccharides
MAFLD	Metabolic-associated fatty liver disease
MRS	de Man, Rogosa and Sharpe (medium)
MTHF	Methyltetrahydrofolate
MTHFR	Methylenetetrahydrofolate reductase
MTT	Microbiota transfer therapy
MTX	Methotrexate
NAFLD	Non-alcoholic fatty liver disease
NOS	Nitric oxide synthase
NTD	Neural tube defects
OCM	One-carbon metabolism
pABA	Para-aminobenzoic acid
PCFT	Proton-coupled folate transporter
PTEN	Phosphatase and tensin homolog
PRISMA	Preferred Reporting Items for Systematic Reviews and Meta-analyses
RBC	Red blood cell
RFC	Reduced folate carrier
RNA	Ribonucleic acid
SAM	S-adenosylmethionine
SCFA	Short-chain fatty acid
T2D	Type II diabetes mellitus
THF	Tetrahydrofolate
UC	Ulcerative colitis
