# Supplementary material for: A Systematic Review of Folate and the Human Enteric Microbiome: Biological Mechanisms and Clinical Implications
Source: Int J Mol Sci. 2026 Jun 3;27(11):5048. doi: 10.3390/ijms27115048 (PMC13257207; doi:10.3390/ijms27115048)
Supplement: Supplementary file 1 [file ijms-27-05048-s001.zip › Table-S2-Combined-GRADE-and-Risk-of-Bias.pdf]

Table S2. Combined GRADE Certainty and Risk-of-Bias Assessment of Included Studies

This combined supplementary table presents, for every included study, the design-appropriate risk-of-bias judgment together with the GRADE certainty rating. Risk of bias was assessed using design-appropriate tools: Cochrane Risk of Bias 2 (RoB 2) for randomized controlled trials; ROBINS-I for non-randomized studies of interventions; the Newcastle-Ottawa Scale (NOS) for observational human studies (cohort, case-control, cross-sectional); SYRCLE's Risk of Bias tool for animal experimental studies; and a structured narrative methodological appraisal for in vitro, in silico, ex vivo, computational, and mechanistic studies. Overall judgments are colour-coded: green = low risk / good quality; yellow = some concerns / fair / moderate; orange = high / serious / critical risk.

Table S2.1. In vitro studies

Ref	Study	Context	Design	Bias tool	Domain-level judgment	Overall RoB	Comment	Starting certainty	Final GRADE
82	Wu et al., 2023	*Lactiplantibacillus plantarum* LZ227; folate-producing strain modulating human gut microbiota	In vitro / fecal fermentation	Narrative QC	Adequate strain characterization; limited replication; ex vivo system	**Moderate**	Single-strain mechanistic study; ecological generalizability limited.	Very low	**Very low**
83	Liu et al., 2022	*Latilactobacillus sakei* LZ217	In vitro / fecal fermentation	Narrative QC	Adequate methodology; limited donor pool	**Moderate**	Donor-specific effects not fully addressed.	Very low	**Very low**
84	Spinler et al., 2014	Human-specific ecotypes of *L. reuteri*	Mechanistic / genomic-functional	Narrative QC	Robust strain comparison; in vitro only	**Moderate**	Strong genomic methods; physiological extrapolation limited.	Very low	**Very low**
85	D'Aimmo et al., 2012	*Bifidobacteria* as natural folate source	In vitro culture	Narrative QC	Reproducible quantification; species-level only	**Moderate**	No ecological context; pure-culture conditions.	Very low	**Very low**
86	Kopp et al., 2017	Intra/extracellular 5-MTHF in *B. adolescentis*	In vitro / analytical	Narrative QC	LC–MS validation; small strain panel	**Low–Moderate**	High analytical rigor; limited strain diversity.	Very low	**Very low**
87	D'Aimmo et al., 2014	Folate biosynthesis across bifidobacteria	In vitro comparative	Narrative QC	Standardized assay; limited functional readouts	**Moderate**	Comparative scope strong; mechanism not tested in host.	Very low	**Very low**
88	Nam et al., 2025	*S. thermophilus* IDCC 2201 / co-culture	In vitro co-culture	Narrative QC	Replicated; single strain	**Moderate**	Co-culture design adds ecological realism.	Very low	**Very low**
97	Nam et al., 2025	*S. thermophilus* IDCC 2201; CRC-relevant mechanism	In vitro + translational	Narrative QC	Mechanism plausible; clinical extrapolation premature	**Moderate**	Translational claims exceed in vitro design.	Very low	**Very low**
98	Soto-Martin et al., 2020	Vitamin biosynthesis cross-feeding in synthetic communities	Synthetic community	Narrative QC	Robust synthetic ecology design; defined consortia	**Low**	Defined-community design strengthens causal inference.	Very low	**Very low**

Ref	Study	Context	Design	Bias tool	Domain-level judgment	Overall RoB	Comment	Starting certainty	Final GRADE
99	Kundra et al., 2024	Folate supports R. intestinalis growth	Fecal batch fermentation	Narrative QC	Replicated; donor-dependent	**Moderate**	Donor variability not controlled by stratification.	Very low	**Very low**
100	Sharma et al., 2019	Folate cycling and ecosystem stability	In vitro mechanistic	Narrative QC	Strong mechanistic design	**Low–Moderate**	Limited replication across donor inocula.	Very low	**Very low**
101	Reigada et al., 2020	*C. elegans*–*E. coli* model; lifespan extension	Mechanistic host-microbe model	Narrative QC	Reproducible model; non-mammalian host	**Moderate**	Generalizability to mammalian gut limited.	Very low	**Very low**
102	Virk et al., 2016	High *E. coli* folate accelerates aging in *C. elegans*	Mechanistic host-microbe model	Narrative QC	Convergent with Reigada et al.	**Moderate**	Same model-system limitations.	Very low	**Very low**
103	Wan et al., 2022	Producer–consumer balance in folate output	Mechanistic community	Narrative QC	Strong ecological framing	**Moderate**	Pure-culture parameters extrapolated to communities.	Very low	**Very low**

Table S2.2. In silico studies

Ref	Study	Context	Design	Bias tool	Domain-level judgment	Overall RoB	Comment	Starting certainty	Final GRADE
35	Engevik et al., 2019	512 enteric genomes; complete folate synthesis in 13%	In silico genomic	Narrative QC	Comprehensive genome panel; standard pipelines	**Low**	Strong genome coverage; limited functional validation.	Very low	**Very low**
81	Magnusdottir et al., 2015	Systematic B-vitamin biosynthesis genome assessment	In silico reconstruction	Narrative QC	Validated reconstruction; well-curated	**Low**	Functional inference assumed from gene presence.	Very low	**Very low**
90	Zhang et al., 2024	Genomic analysis of *L. plantarum* ZFM55	In silico + *in vitro*	Narrative QC	Mixed methods; single strain	**Moderate**	Limited generalizability beyond strain.	Very low	**Very low**
91	Saulnier et al., 2011	Pathway reconstruction in *L. reuteri*	In silico / transcriptomic	Narrative QC	Pathway and expression data congruent	**Low–Moderate**	Cross-validated transcriptomics.	Very low	**Very low**
92	Kachi et al., 2024	Novel folate-pathway enzyme in *L. reuteri*	Mechanistic molecular	Narrative QC	Replicated enzymatic characterization	**Low**	Strong biochemical validation.	Very low	**Very low**
93	Mazhar et al., 2022	*Bacillus subtilis* DE111 properties	In vitro + *in silico*	Narrative QC	Multi-method; commercial-strain context	**Moderate**	Sponsorship and selection effects possible.	Very low	**Very low**
94	Kapse et al., 2018	*Bacillus clausii* B106 genome	In silico profiling	Narrative QC	Standard genomic methods	**Moderate**	Single-genome analysis.	Very low	**Very low**

Ref	Study	Context	Design	Bias tool	Domain-level judgment	Overall RoB	Comment	Starting certainty	Final GRADE
95	Elkalla et al., 2024	*P. acidilactici* WNYM01/02 genomes	In silico characterization	Narrative QC	Genome quality reported	**Moderate**	Functional inference not validated in vitro.	Very low	**Very low**
96	Aziz et al., 2025	Mucosal LAB probiotic potential	In silico + phenotypic	Narrative QC	Mixed methods; limited phenotypic validation	**Moderate**	Probiotic claims not host-tested.	Very low	**Very low**
104	Rodionov et al., 2019	2,228 genomes / 690 species; B-vitamin phenotypes	In silico subsystem reconstruction	Narrative QC	Comprehensive validated pipeline	**Low**	Highest-quality reference dataset for the field.	Very low	**Very low**
105	Jiang et al., 2022	Ruminant metagenomics across species	In silico metagenomic	Narrative QC	Solid metagenomic methods	**Moderate**	Host-species generalizability limited.	Very low	**Very low**
106	Hillman et al., 2020	*R. inulinivorans* folate evidence	Comparative genomics	Narrative QC	Specific-genus focus; limited validation	**Moderate**	In silico inference only.	Very low	**Very low**
107	Dos Anjos Almeida et al., 2025	Poultry/swine Lactobacillales B-vitamin pathways	Comparative genomics	Narrative QC	Standard pipeline; livestock focus	**Moderate**	Translational relevance to humans uncertain.	Very low	**Very low**

Table S2.3. Normal health studies

Ref	Study	Context	Design	Bias tool	Domain-level judgment	Overall RoB	Comment	Starting certainty	Final GRADE
108	Radjabzadeh et al., 2020	Dutch children/adults; age-related folate signatures	Cross-sectional human cohort	NOS (cross-sectional)	Selection 3/4; Comparability 1/2; Outcome 2/3	**7 stars — Good**	Large population sample; limited adjustment for diet.	Low	**Low**
109	Malinowska et al., 2022	Folate production vs alpha diversity	Cross-sectional + culture	NOS (cross-sectional)	Selection 2/4; Comparability 1/2; Outcome 2/3	**5 stars — Fair**	Single-cohort correlational design.	Low	**Low**
110	Kulecka et al., 2020	Endurance athletes vs sedentary controls	Case-control human	NOS (case-control)	Selection 3/4; Comparability 1/2; Exposure 2/3	**6 stars — Fair**	Convenience sampling; matching incomplete.	Low	**Low**
111	Jiang et al., 2022	6-month voyage; longitudinal folate biosynthesis	Longitudinal observational	NOS (cohort)	Selection 3/4; Comparability 1/2; Outcome 2/3	**6 stars — Fair**	Small specialized cohort; environmental confounding.	Low	**Low**
112	Jarmukhanov et al., 2024	Frailty severity and folate biosynthesis	Cross-sectional	NOS (cross-sectional)	Selection 3/4; Comparability 1/2; Outcome 2/3	**6 stars — Fair**	Single-center recruitment; limited covariate adjustment.	Low	**Low**

Table S2.4. Metabolic disease studies

Ref	Study	Context	Design	Bias tool	Domain-level judgment	Overall RoB	Comment	Starting certainty	Final GRADE
113	Köse et al., 2020	Serum/RBC folate and obesity risk	Human observational	NOS (cross-sectional)	Selection 3/4; Comparability 1/2; Outcome 2/3	**6 stars — Fair**	Single-cohort; dietary intake self-reported.	Low	**Low**
114	Riggen-Bueno et al., 2024	Obesity-associated diversity & folate metabolism	Observational metagenomic	NOS (cross-sectional)	Selection 3/4; Comparability 1/2; Outcome 2/3	**6 stars — Fair**	Inferred-function risk.	Low	**Low**
115	Hu et al., 2022	*Parasutterella*, *Butyrivibrio*, Clostridium correlations	Observational	NOS (cross-sectional)	Selection 3/4; Comparability 1/2; Outcome 2/3	**6 stars — Fair**	Cross-sectional inference.	Low	**Low**
116	Santacruz et al., 2010	Depleted *Bacteroides*/*Bifidobacterium* correlation	Observational	NOS (cross-sectional)	Selection 3/4; Comparability 1/2; Outcome 2/3	**6 stars — Fair**	Older sequencing methods limit precision.	Low	**Low**
117	Hasebe et al., 2025	Folic acid intake and phyla shifts	Observational + animal	NOS / SYRCLE (mixed)	Mixed-design; conservative judgment	**Fair / Moderate**	Mixed methods limit single-tool rating.	Low	**Low**
118	Sample et al., 2025	Folate biosynthesis in obesity multi-omics	Observational multi-omics	NOS (cross-sectional)	Selection 3/4; Comparability 2/2; Outcome 2/3	**7 stars — Good**	Multi-omic strengthens internal validity.	Low	**Low**
119	Rabbani et al., 2025	MASLD pathophysiology	Background observational/experimental	Narrative QC	Mechanism-oriented review/secondary	**Moderate**	Mixed primary/review elements.	Low	**Low**
120	Sid et al., 2017	Lower folate and MASLD prevalence	Observational	NOS (cross-sectional)	Selection 3/4; Comparability 1/2; Outcome 2/3	**6 stars — Fair**	Self-reported intake; cross-sectional.	Low	**Low**
121	Yang et al., 2025	One-carbon metabolism in steatosis	Mechanistic translational	Narrative QC	Mixed mechanistic methods	**Moderate**	Limited human controls.	Very low	**Very low**
122	Mascardi et al., 2021	Steatosis vs steatohepatitis metatranscriptomic	Observational	NOS (case-control)	Selection 3/4; Comparability 1/2; Exposure 2/3	**6 stars — Fair**	Modest sample size.	Low	**Low**
123	Hu et al., 2025	Vitamin-B pathways and taxa changes	Observational	NOS (cross-sectional)	Selection 3/4; Comparability 1/2; Outcome 2/3	**6 stars — Fair**	Inferred-function design.	Low	**Low**
124	Sun et al., 2024	Reduced folate metabolism with steatosis	Animal	SYRCLE	Sequence allocation unclear; baseline matched; blinding unclear	**Moderate**	Standard reporting gaps for blinding/allocation.	Very low	**Very low**

Ref	Study	Context	Design	Bias tool	Domain-level judgment	Overall RoB	Comment	Starting certainty	Final GRADE
125	Williams et al., 2025	Lower protective taxa & folate pathways	Case-control metagenomic	NOS (case-control)	Selection 3/4; Comparability 2/2; Exposure 2/3	**7 stars — Good**	Well-matched controls strengthen comparability.	Low	**Low**
126	Yanavich et al., 2022	Decreased folate biosynthesis & fibrosis	Observational	NOS (cross-sectional)	Selection 3/4; Comparability 1/2; Outcome 2/3	**6 stars — Fair**	Limited multivariable adjustment.	Low	**Low**
127	Righetti et al., 2025	Severity tied to predicted folate production	Observational	NOS (cross-sectional)	Selection 3/4; Comparability 1/2; Outcome 2/3	**6 stars — Fair**	Inferred-function design.	Low	**Low**
128	Ballesteros et al., 2024	Distinct dysbiosis with metabolic shifts	Observational	NOS (cross-sectional)	Selection 3/4; Comparability 1/2; Outcome 2/3	**6 stars — Fair**	Standard observational limitations.	Low	**Low**
129	Banday et al., 2020	Folate status and diabetes risk	Observational	NOS (cross-sectional)	Selection 3/4; Comparability 1/2; Outcome 2/3	**6 stars — Fair**	Standard observational limitations.	Low	**Low**
130	Mokhtari et al., 2023	Downregulation of folate biosynthesis	Observational	NOS (cross-sectional)	Selection 3/4; Comparability 1/2; Outcome 2/3	**6 stars — Fair**	Inferred-function design.	Low	**Low**
131	Xia et al., 2025	T2D mouse; folate biosynthesis pathways	Animal intervention	SYRCLE	Random allocation reported; blinding unclear	**Moderate**	Typical preclinical reporting limits.	Very low	**Very low**
132	Huang et al., 2022	T2D mouse; folate biosynthesis	Animal intervention	SYRCLE	Allocation method unclear; outcome assessor blinding unclear	**Moderate**	Typical preclinical reporting limits.	Very low	**Very low**

Table S2.5. Gastrointestinal disorder studies

Ref	Study	Context	Design	Bias tool	Domain-level judgment	Overall RoB	Comment	Starting certainty	Final GRADE
133	Adolph et al., 2022	IBD metabolic framing	Background/observational	Narrative QC	Review-secondary content	**Moderate**	Review/secondary use.	Low	**Low**
134	Pan et al., 2017	Reduced serum folate in IBD	Observational/meta-analytic	NOS / Meta	Selection 3/4; Comparability 1/2; Outcome 2/3	**6 stars — Fair**	Heterogeneity across pooled studies.	Low	**Low**
135	Bharali et al., 2025	Future UC risk and folate pathways	Prospective cohort	NOS (cohort)	Selection 4/4; Comparability 2/2; Outcome 2/3	**8 stars — Good**	Prospective design strengthens causality.	Low	**Low**
136	Xu et al., 2016	Low serum folate, lower fecal *Lactobacillus*, SCFA	Animal	SYRCLE	Allocation/blinding poorly described	**Moderate**	Typical preclinical reporting limits.	Very low	**Very low**

Ref	Study	Context	Design	Bias tool	Domain-level judgment	Overall RoB	Comment	Starting certainty	Final GRADE
137	Klaseen et al., 2019	Depletion of microbial folate biosynthesis genes	Observational	NOS (case-control)	Selection 3/4; Comparability 1/2; Exposure 2/3	**6 stars — Fair**	Inferred-function design.	Low	**Low**
138	Lima et al., 2024	Sulfasalazine & *F. prausnitzii*	Mixed observational/*in vitro*	Mixed (NOS + Narrative)	Mixed-design judgment	**Moderate**	Combined methodologies limit single-tool grading.	Low	**Low**
139	Haskey et al., 2025	12-week diet altered folate biosynthesis pathways	Human dietary RCT	RoB 2	D1 Low; D2 Some concerns (open-label); D3 Low; D4 Some concerns; D5 Low	**Some concerns**	Open-label diet trial; objective microbial outcomes mitigate.	High	**Moderate**
140	Kim et al., 2026	Folate malabsorption & supplementation in gastritis	Mixed observational/intervention	ROBINS-I	Confounding Moderate; selection Moderate; classification Low; deviations Moderate; missing Low; measurement Low; reporting Low	**Moderate**	Non-randomized intervention component.	Low	**Low**
141	Sung et al., 2020	Atrophy/metaplasia and microbial folate biosynthesis	Observational	NOS (cross-sectional)	Selection 3/4; Comparability 1/2; Outcome 2/3	**6 stars — Fair**	Standard observational limitations.	Low	**Low**
142	Quinn et al., 2019	Dietary folate & antibiotics in gastric pathology	Animal intervention	SYRCLE	Allocation reported; blinding unclear	**Moderate**	Typical preclinical reporting limits.	Very low	**Very low**
143	Wielgosz-Grochowska et al., 2024	Elevated serum folate in SIBO subtype	Observational	NOS (cross-sectional)	Selection 3/4; Comparability 1/2; Outcome 2/3	**6 stars — Fair**	Subtype stratification reduces baseline comparability.	Low	**Low**
144	Platovsky et al., 2014	SIBO and elevated serum folate	Observational	NOS (cross-sectional)	Selection 3/4; Comparability 1/2; Outcome 2/3	**6 stars — Fair**	Older diagnostic criteria for SIBO.	Low	**Low**
145	Guo et al., 2024	Reduced one-carbon pool despite higher host folate	Observational metagenomic	NOS (cross-sectional)	Selection 3/4; Comparability 1/2; Outcome 2/3	**6 stars — Fair**	Inferred-function design.	Low	**Low**
146	Weng et al., 2023	IBS-like phenotype, lower blood folate	Animal	SYRCLE	Allocation/blinding unclear	**Moderate**	Typical preclinical reporting limits.	Very low	**Very low**

Table S2.6. Cancer studies

Ref	Study	Context	Design	Bias tool	Domain-level judgment	Overall RoB	Comment	Starting certainty	Final GRADE
147	Wang et al., 2026	17 co-metabolites in CRC communities	Computational	Narrative QC	Modeling assumptions critical; no in vivo validation	**Moderate**	Computational only.	Very low	**Very low**
148	Liss et al., 2018	Prostate cancer fecal microbiome	Case-control	NOS (case-control)	Selection 3/4; Comparability 1/2; Exposure 2/3	**6 stars — Fair**	Modest sample size; selection from clinic.	Low	**Low**
149	Wakamori et al., 2024	Prostate cancer in mice and humans	Mixed human + animal	Mixed (NOS + SYRCLE)	Mixed-design judgment	**Moderate**	Animal arm dominant; human arm small.	Low	**Low**
150	Muratore et al., 2025	Non-cancer microbiome with enhanced folate	Case-control	NOS (case-control)	Selection 3/4; Comparability 1/2; Exposure 2/3	**6 stars — Fair**	Standard case-control limitations.	Low	**Low**
151	Yoon et al., 2023	Decreased folate biosynthesis in lymphoma	Case-control	NOS (case-control)	Selection 3/4; Comparability 1/2; Exposure 2/3	**6 stars — Fair**	Treatment exposure may confound.	Low	**Low**
152	Byrd et al., 2018	Cancer status and folate biosynthesis	Observational	NOS (cross-sectional)	Selection 3/4; Comparability 1/2; Outcome 2/3	**6 stars — Fair**	Inferred-function design.	Low	**Low**

Table S2.7. Psychiatric disease studies

Ref	Study	Context	Design	Bias tool	Domain-level judgment	Overall RoB	Comment	Starting certainty	Final GRADE
153	Komijani et al., 2025	ASD vs controls; taxa differences	Case-control	NOS (case-control)	Selection 3/4; Comparability 1/2; Exposure 2/3	**6 stars — Fair**	Diet/medication confounding common in ASD.	Low	**Low**
154	Ling et al., 2022	Higher microbiota folate biosynthesis in schizophrenia	Case-control	NOS (case-control)	Selection 3/4; Comparability 1/2; Exposure 2/3	**6 stars — Fair**	Antipsychotic exposure incompletely controlled.	Low	**Low**
155	Miao et al., 2021	Reduced *Bifidobacteria* & serum folate	Case-control	NOS (case-control)	Selection 3/4; Comparability 1/2; Exposure 2/3	**6 stars — Fair**	Modest sample; medication effects.	Low	**Low**
156	Dai et al., 2025	Decreased alpha diversity & folate disturbance	Observational	NOS (cross-sectional)	Selection 3/4; Comparability 1/2; Outcome 2/3	**6 stars — Fair**	Cross-sectional inference.	Low	**Low**

Table S2.8. Cardiovascular disease studies

Ref	Study	Context	Design	Bias tool	Domain-level judgment	Overall RoB	Comment	Starting certainty	Final GRADE
157	Liu et al., 2020	Controls enriched in folate-transforming taxa	Cohort/case-control	NOS (cohort)	Selection 3/4; Comparability 2/2; Outcome 2/3	**7 stars — Good**	Reasonable matching reported.	Low	**Low**
158	Ai et al., 2024	Reduced predicted microbial folate biosynthesis	Case-control	NOS (case-control)	Selection 3/4; Comparability 1/2; Exposure 2/3	**6 stars — Fair**	Inferred-function design.	Low	**Low**
159	Park et al., 2021	Downregulated thiamine and folate biosynthesis	Observational	NOS (cross-sectional)	Selection 3/4; Comparability 1/2; Outcome 2/3	**6 stars — Fair**	Inferred-function design.	Low	**Low**

Table S2.9. Neurologic disease studies

Ref	Study	Context	Design	Bias tool	Domain-level judgment	Overall RoB	Comment	Starting certainty	Final GRADE
160	Park et al., 2022	Folate/B12 deficiency and memory	Animal	SYRCLE	Allocation/blinding unclear	**Moderate**	Typical preclinical reporting.	Very low	**Very low**
161	An et al., 2024	*Bifidobacterium*/*Lachnospiraceae* mediate B-vitamin/AD risk	Genetic epidemiology / Mendelian Randomization	NOS-adapted	Selection 3/4; Comparability 2/2; Outcome 2/3	**7 stars — Good**	MR design strengthens causal inference.	Low	**Low**
162	Rosario et al., 2021	Reduced microbial capacity for folate	Observational multi-omics	NOS (cross-sectional)	Selection 3/4; Comparability 1/2; Outcome 2/3	**6 stars — Fair**	Multi-omics strengthens internal validity.	Low	**Low**
163	Chen et al., 2024	Folate intake & microbial diversity & sleep	Observational	NOS (cross-sectional)	Selection 3/4; Comparability 1/2; Outcome 2/3	**6 stars — Fair**	Self-reported intake.	Low	**Low**

Table S2.10. Immune disorder studies

Ref	Study	Context	Design	Bias tool	Domain-level judgment	Overall RoB	Comment	Starting certainty	Final GRADE
164	Wang et al., 2022	Folate enriched in plaque vs guttate psoriasis	Observational	NOS (case-control)	Selection 3/4; Comparability 1/2; Exposure 2/3	**6 stars — Fair**	Subtype-stratified design.	Low	**Low**
165	Mirzaei et al., 2024	Shift away from folate-producing communities	Observational/mechanistic	NOS (cross-sectional)	Selection 3/4; Comparability 1/2; Outcome 2/3	**6 stars — Fair**	Mixed methods; sample size limited.	Low	**Low**

Ref	Study	Context	Design	Bias tool	Domain-level judgment	Overall RoB	Comment	Starting certainty	Final GRADE
166	Wang et al., 2024	Sepsis; serum folate-*Sellimonas* correlation	Observational	NOS (cross-sectional)	Selection 3/4; Comparability 1/2; Outcome 2/3	**6 stars — Fair**	ICU population; treatment heterogeneity.	Low	**Low**
167	Ta et al., 2020	Infant eczema trajectory and folate biosynthesis	Longitudinal cohort	NOS (cohort)	Selection 4/4; Comparability 2/2; Outcome 2/3	**8 stars — Good**	Prospective design strengthens inference.	Low	**Low**
168	Mohandas et al., 2018	High-risk children; folic acid and diversity	Cross-sectional inpatient	NOS (cross-sectional)	Selection 3/4; Comparability 1/2; Outcome 2/3	**6 stars — Fair**	Inpatient setting; multiple comorbidities.	Low	**Low**

Table S2.11. Female reproduction studies

Ref	Study	Context	Design	Bias tool	Domain-level judgment	Overall RoB	Comment	Starting certainty	Final GRADE
169	Rhoades et al., 2022	Macaque pregnancy/postpartum microbiome	Animal longitudinal	SYRCLE-adapted	Adequate longitudinal sampling; blinding unclear	**Moderate**	Strong design for nonhuman primate; reporting gaps.	Very low	**Very low**
117	Hasebe et al., 2025	Maternal folate status and *Lactobacillus/Romboutsia*	Animal	SYRCLE	Allocation/blinding unclear	**Moderate**	Typical preclinical reporting.	Very low	**Very low**
170	Yu et al., 2025	Preeclampsia; lower RBC folate forms	Case-control	NOS (case-control)	Selection 3/4; Comparability 2/2; Exposure 2/3	**7 stars — Good**	Reasonable matching.	Low	**Low**
171	Lv et al., 2022	Early-onset preeclampsia metagenomics	Case-control metagenomic	NOS (case-control)	Selection 3/4; Comparability 1/2; Exposure 2/3	**6 stars — Fair**	Standard case-control limitations.	Low	**Low**

Table S2.12. Probiotic intervention studies

Ref	Study	Context	Design	Bias tool	Domain-level judgment	Overall RoB	Comment	Starting certainty	Final GRADE
172	Zhang et al., 2020	Folate-biofortified yogurt in folate-deficient rats	Animal controlled	SYRCLE	Random allocation reported; blinding unclear	**Moderate**	Typical preclinical reporting.	Very low	**Very low**
173	Thomas et al., 2016	*L. reuteri* folate suppresses inflammation	Mechanistic/animal-linked	SYRCLE-adapted	Mixed in vitro/in vivo	**Moderate**	Mixed-design judgment.	Very low	**Very low**
174	Li et al., 2024	*B. thetaiotaomicron* in mouse steatosis	Animal controlled	SYRCLE	Allocation reported; blinding unclear	**Moderate**	Typical preclinical reporting.	Very low	**Very low**

Ref	Study	Context	Design	Bias tool	Domain-level judgment	Overall RoB	Comment	Starting certainty	Final GRADE
175	Qiao et al., 2025	Cross-feeding probiotic combination	Animal controlled	SYRCLE	Allocation reported; blinding unclear	**Moderate**	Typical preclinical reporting.	Very low	**Very low**
176	Jana et al., 2025	*Phytobacter* sp. RSE02 in HFD mice	Animal controlled	SYRCLE	Allocation reported; blinding unclear	**Moderate**	Typical preclinical reporting.	Very low	**Very low**
177	Ma et al., 2024	Probiotic in pregnancy; healthy women	Human RCT	RoB 2	D1 Low; D2 Low; D3 Low; D4 Low; D5 Some concerns	**Low**	Healthy-volunteer RCT; selective reporting risk small.	High	**Moderate**
178	Ma et al., 2025	Maternal pregnancy probiotic; infant follow-up	Prospective interventional follow-up	ROBINS-I	Confounding Moderate; selection Low; classification Low; deviations Moderate; missing Moderate; measurement Low; reporting Low	**Moderate**	Non-randomized follow-up arm.	Low	**Low**
179	Peng et al., 2024	*A. muciniphila* in mouse preeclampsia	Animal controlled	SYRCLE	Allocation reported; blinding unclear	**Moderate**	Typical preclinical reporting.	Very low	**Very low**
180	Nirmalkar et al., 2022	MTT in children with ASD	Open-label before-after	ROBINS-I	Confounding High; selection Low; classification Low; deviations High; missing Low; measurement Moderate; reporting Low	**Serious**	Open-label, no concurrent control; placebo effects substantial.	Low	**Very low**
181	Valentini et al., 2015	RISTOMED RCT in older adults	Human RCT	RoB 2	D1 Low; D2 Some concerns (open-label); D3 Low; D4 Low; D5 Low	**Some concerns**	Open-label diet/probiotic trial.	High	**Moderate**
182	Ke et al., 2025	Probiotic-fermented milk in preeclampsia rats	Animal controlled	SYRCLE	Allocation reported; blinding unclear	**Moderate**	Typical preclinical reporting.	Very low	**Very low**
183	Nybroe et al., 2022	Synbiotic with *E. faecium* in dogs	Animal crossover	SYRCLE-adapted	Crossover design; allocation unclear	**Moderate**	Within-subject design strengthens inference; reporting gaps.	Very low	**Very low**

Table S2.13. Prebiotic intervention studies

Ref	Study	Context	Design	Bias tool	Domain-level judgment	Overall RoB	Comment	Starting certainty	Final GRADE
131	Xia et al., 2025	T2D mouse model	Animal	SYRCLE	Allocation reported; blinding unclear	**Moderate**	Typical preclinical reporting.	Very low	**Very low**
132	Huang et al., 2022	T2D mouse model	Animal	SYRCLE	Allocation reported; blinding unclear	**Moderate**	Typical preclinical reporting.	Very low	**Very low**
184	Li et al., 2025	Human prebiotic trial	Human RCT	RoB 2	D1 Low; D2 Low; D3 Low; D4 Low; D5 Low	**Low**	Adequately reported RCT.	High	**Moderate**
185	Lan et al., 2026	Human prebiotic trial	Human RCT	RoB 2	D1 Low; D2 Some concerns; D3 Low; D4 Low; D5 Low	**Some concerns**	Allocation concealment unclear.	High	**Moderate**
186	Zhou et al., 2023	Human prebiotic trial	Human RCT	RoB 2	D1 Some concerns; D2 Some concerns; D3 Low; D4 Low; D5 Low	**Some concerns**	Randomization method incompletely reported.	High	**Low**
187	Fava et al., 2022	Human prebiotic trial	Human RCT	RoB 2	D1 Low; D2 Some concerns; D3 Low; D4 Low; D5 Low	**Some concerns**	Open-label dietary intervention.	High	**Low**
188	Yaskolka Meir et al., 2021	Human prebiotic trial	Human RCT	RoB 2	D1 Low; D2 Some concerns; D3 Low; D4 Low; D5 Low	**Some concerns**	Open-label dietary intervention.	High	**Low**
189	Turker et al., 2017	Human prebiotic trial	Human RCT	RoB 2	D1 Some concerns; D2 Some concerns; D3 Low; D4 Some concerns; D5 Low	**Some concerns**	Older trial; allocation/blinding incompletely reported.	High	**Low**
190	Cui et al., 2026	HFD dysbiosis and hepatic folate pathways	Animal	SYRCLE	Allocation reported; blinding unclear	**Moderate**	Typical preclinical reporting.	Very low	**Very low**
191	Qiao et al., 2020	Folate-producing *Bacteroides* enrichment	Animal	SYRCLE	Allocation reported; blinding unclear	**Moderate**	Typical preclinical reporting.	Very low	**Very low**
192	Wang et al., 2024	Increased colonic folate	Animal	SYRCLE	Allocation reported; blinding unclear	**Moderate**	Typical preclinical reporting.	Very low	**Very low**
193	Liu et al., 2021	Upregulated folate biosynthesis enzyme pathway	Animal	SYRCLE	Allocation reported; blinding unclear	**Moderate**	Typical preclinical reporting.	Very low	**Very low**
194	Bajic et al., 2023	Age-dependent *ex vivo* folate	Ex vivo human microbiota	Narrative QC	Multi-donor design; ex vivo only	**Moderate**	Donor variability addressed; mechanism not host-tested.	Very low	**Very low**

Ref	Study	Context	Design	Bias tool	Domain-level judgment	Overall RoB	Comment	Starting certainty	Final GRADE
195	Huang et al., 2026	Altered fecal and serum folate	Animal	SYRCLE	Allocation reported; blinding unclear	**Moderate**	Typical preclinical reporting.	Very low	**Very low**
196	Qu et al., 2023	Altered predicted folate synthesis	Animal	SYRCLE	Allocation reported; blinding unclear	**Moderate**	Typical preclinical reporting.	Very low	**Very low**

Table S2.14. Dietary intervention studies

Ref	Study	Context	Design	Bias tool	Domain-level judgment	Overall RoB	Comment	Starting certainty	Final GRADE
197	Palladino et al., 2021	Circulating folate and transporter expression	Animal dietary	SYRCLE	Allocation reported; blinding unclear	**Moderate**	Typical preclinical reporting.	Very low	**Very low**
117	Hasebe et al., 2025	Maternal/offspring folate; microbiota shifts	Animal dietary	SYRCLE	Allocation reported; blinding unclear	**Moderate**	Typical preclinical reporting.	Very low	**Very low**
139	Haskey et al., 2025	12-week diet altered folate biosynthesis	Human dietary RCT	RoB 2	D1 Low; D2 Some concerns (open-label); D3 Low; D4 Some concerns; D5 Low	**Some concerns**	Open-label diet trial; objective outcomes mitigate.	High	**Moderate**
198	Carroccio et al., 2021	Crossover/parallel diet study	Human crossover/parallel	RoB 2	D1 Some concerns; D2 Some concerns; D3 Low; D4 Some concerns; D5 Low	**Some concerns**	Open-label; blinding limited.	High	**Low**

Table S2.15. Folate intervention studies

Ref	Study	Context	Design	Bias tool	Domain-level judgment	Overall RoB	Comment	Starting certainty	Final GRADE
199	Liu et al., 2023	Broiler/laying hen microbiota	Animal	SYRCLE	Allocation/blinding poorly described	**Moderate**	Typical livestock-study reporting.	Very low	**Very low**
200	Bai et al., 2021	Broiler/laying hen microbiota	Animal	SYRCLE	Allocation/blinding poorly described	**Moderate**	Typical livestock-study reporting.	Very low	**Very low**
201	Zhang et al., 2022	Broiler/laying hen microbiota	Animal	SYRCLE	Allocation/blinding poorly described	**Moderate**	Typical livestock-study reporting.	Very low	**Very low**

Ref	Study	Context	Design	Bias tool	Domain-level judgment	Overall RoB	Comment	Starting certainty	Final GRADE
202	Li et al., 2024	Broiler/laying hen microbiota	Animal	SYRCLE	Allocation/blinding poorly described	**Moderate**	Typical livestock-study reporting.	Very low	**Very low**
203	Cordero-Varela et al., 2023	Folate supplementation and SCFA changes	Animal	SYRCLE	Allocation/blinding poorly described	**Moderate**	Typical preclinical reporting.	Very low	**Very low**
204	Wang et al., 2019	Cellulolytic bacteria and protein synthesis	Animal	SYRCLE	Allocation/blinding poorly described	**Moderate**	Typical preclinical reporting.	Very low	**Very low**
205	Wang et al., 2016	Cellulolytic bacteria and protein synthesis	Animal	SYRCLE	Allocation/blinding poorly described	**Moderate**	Typical preclinical reporting.	Very low	**Very low**
206	Liu et al., 2020	Increased cellulolytic bacteria	Animal	SYRCLE	Allocation/blinding poorly described	**Moderate**	Typical preclinical reporting.	Very low	**Very low**
16	Wang et al., 2021	Increased *Lactobacillus* spp. and SCFAs	Animal	SYRCLE	Allocation/blinding poorly described	**Moderate**	Typical preclinical reporting.	Very low	**Very low**
207	Jiao et al., 2020	Folate vs synthetic folic acid	Mixed translational	Mixed (RoB 2 / SYRCLE)	Mixed-design judgment	**Moderate–Some concerns**	Mixed-design limits single-tool grade.	Low	**Low**
208	Zinno et al., 2020	Folate vs synthetic folic acid	Mixed translational	Mixed (RoB 2 / SYRCLE)	Mixed-design judgment	**Moderate–Some concerns**	Mixed-design limits single-tool grade.	Low	**Low**
209	Zheng et al., 2024	Community composition and SCFA	Ex vivo / *in vitro*	Narrative QC	Adequate methodology; limited replicates	**Moderate**	Ex vivo only.	Very low	**Very low**
41	Maynard et al., 2018	Indirect host benefit via bacterial folate	Mechanistic host-microbe	Narrative QC	Strong mechanistic design	**Low–Moderate**	Strong mechanistic inference.	Very low	**Very low**
17	Wang et al., 2023	Maternal/offspring microbiota	Animal	SYRCLE	Allocation/blinding poorly described	**Moderate**	Typical preclinical reporting.	Very low	**Very low**
203	Cordero-Varela et al., 2023	Uric acid and microbiota	Mixed	Mixed	Mixed-design judgment	**Moderate**	Mixed methods.	Low	**Low**
210	Sun et al., 2022	Uric acid and microbiota	Mixed	Mixed	Mixed-design judgment	**Moderate**	Mixed methods.	Low	**Low**
211	Wang et al., 2022	Reduced H2S-producing Clostridiales	Animal	SYRCLE	Allocation/blinding poorly described	**Moderate**	Typical preclinical reporting.	Very low	**Very low**

Ref	Study	Context	Design	Bias tool	Domain-level judgment	Overall RoB	Comment	Starting certainty	Final GRADE
212	Chen et al., 2022	Anti-obesity effects depend on microbiome	Animal	SYRCLE	Allocation reported; blinding unclear	**Moderate**	Typical preclinical reporting.	Very low	**Very low**
213	Han et al., 2023	Anti-obesity effects depend on microbiome	Animal	SYRCLE	Allocation reported; blinding unclear	**Moderate**	Typical preclinical reporting.	Very low	**Very low**
214	Zhang et al., 2023	Offspring microbiota and hepatic inflammation	Animal	SYRCLE	Allocation reported; blinding unclear	**Moderate**	Typical preclinical reporting.	Very low	**Very low**
215	Molinero et al., 2025	Increased *Bifidobacterium* and *Lactobacillus*	Human intervention	ROBINS-I	Confounding Moderate; selection Low; classification Low; deviations Moderate; missing Low; measurement Low; reporting Low	**Moderate**	Non-randomized human trial.	High	**Low**

Table S2.16. Other intervention studies

Ref	Study	Context	Design	Bias tool	Domain-level judgment	Overall RoB	Comment	Starting certainty	Final GRADE
216	Deng et al., 2024	Restored 7,8-dihydropteroic acid	Animal	SYRCLE	Allocation reported; blinding unclear	**Moderate**	Typical preclinical reporting.	Very low	**Very low**
217	Bhalla et al., 2022	Increased extracellular folate in *E. durans*	Computational + *in vitro*	Narrative QC	Mixed methods; reproducibility adequate	**Moderate**	Computational + in vitro only.	Very low	**Very low**
